# The host-directed therapeutic imatinib mesylate accelerates immune responses to *Mycobacterium marinum* infection and limits pathology associated with granulomas

**DOI:** 10.1371/journal.ppat.1011387

**Published:** 2023-05-18

**Authors:** Tesia L. Cleverley, Siri Peddineni, Jeannette Guarner, Francesca Cingolani, Pamela K. Garcia, Heather Koehler, Edward S. Mocarski, Daniel Kalman

**Affiliations:** 1 Department of Pathology and Laboratory Medicine, Emory University School of Medicine, Atlanta, Georgia, United States of America; 2 Immunology and Molecular Pathogenesis Graduate Program, Emory University School of Medicine, Atlanta, Georgia, United States of America; 3 Department of Microbiology and Immunology, Emory University School of Medicine, Atlanta, Georgia, United States of America; Portland VA Medical Center, Oregon Health and Science University, UNITED STATES

## Abstract

Infections caused by members of the mycobacterium tuberculosis complex [MTC] and nontuberculous mycobacteria [NTM] can induce widespread morbidity and mortality in people. Mycobacterial infections cause both a delayed immune response, which limits rate of bacterial clearance, and formation of granulomas, which contain bacterial spread, but also contribute to lung damage, fibrosis, and morbidity. Granulomas also limit access of antibiotics to bacteria, which may facilitate development of resistance. Bacteria resistant to some or all antibiotics cause significant morbidity and mortality, and newly developed antibiotics readily engender resistance, highlighting the need for new therapeutic approaches. Imatinib mesylate, a cancer drug used to treat chronic myelogenous leukemia [CML] that targets Abl and related tyrosine kinases, is a possible host-directed therapeutic [HDT] for mycobacterial infections, including those causing TB. Here, we use the murine *Mycobacterium marinum* [Mm] infection model, which induces granulomatous tail lesions. Based on histological measurements, imatinib reduces both lesion size and inflammation of surrounding tissue. Transcriptomic analysis of tail lesions indicates that imatinib induces gene signatures indicative of immune activation and regulation at early time points post infection that resemble those seen at later ones, suggesting that imatinib accelerates but does not substantially alter anti-mycobacterial immune responses. Imatinib likewise induces signatures associated with cell death and promotes survival of bone marrow-derived macrophages [BMDMs] in culture following infection with Mm. Notably, the capacity of imatinib to limit formation and growth of granulomas *in vivo* and to promote survival of BMDMs *in vitro* depends upon caspase 8, a key regulator of cell survival and death. These data provide evidence for the utility of imatinib as an HDT for mycobacterial infections in accelerating and regulating immune responses, and limiting pathology associated with granulomas, which may mitigate post-treatment morbidity.

## Author summary

Mycobacterial infections remain an important cause of morbidity and mortality in humans; for example, *Mycobacterium tuberculosis* [Mtb], the cause of tuberculosis [TB], kills ~1.5 million and newly infects ~10 million each year. Although most people effectively combat mycobacterial infections, treatment is compromised in at-risk individuals by an indolent immune response and chronic inflammation, which results in granulomas that encase the bacteria and limit spread. Granulomas also contribute to tissue damage and limit access of antibiotics to bacteria, which engenders resistance. We proposed using imatinib mesylate, a host directed therapeutic [HDT], against mycobacterial infections. Imatinib, a cancer therapeutic that inhibits Abl and related tyrosine kinases, alters intracellular transit of bacteria during infection. Using systems biology approaches in conjunction with murine infections with *Mycobacterium marinum*, a close genetic relative of Mtb that forms tail granulomas, we report that imatinib does not fundamentally alter the anti-mycobacteria immune response, but rather accelerates it. In addition, imatinib regulates gene signatures associated with cell death, and limits granuloma formation and growth, an effect abrogated in mice lacking caspase 8, a key regulator of cell survival and death. These data highlight imatinib as a possible HDT for mycobacterial infections, including TB, with the capacity to augment the immune response in at-risk individuals, and limit granuloma growth, thereby limiting tissue damage and post-treatment morbidity.

## Introduction

Pathogenic mycobacteria have developed numerous strategies to manipulate and evade host immune responses. Notable members of this genus include *Mycobacterium tuberculosis* [Mtb], the causative agent of tuberculosis [TB], a leading cause of morbidity and mortality that claims 1.5 million lives each year [1], *Mycobacterium leprae*, which causes Hansen’s disease (leprosy), *Mycobacterium avium- intracellulare*, an opportunistic pathogen affecting immunocompromised patients and those with severe lung disease such as cystic fibrosis, and *Mycobacterium marinum* [Mm], a human pathogen acquired from contaminated aqueous environments or infected fish that causes skin lesions called “fish tank granulomas” [2]. All these bacteria are either naturally resistant to antibiotics or readily acquire resistance [3,4]. The standard of care for TB, for example, is a multi-drug antibiotic regimen given over four to nine months. Importantly, Mtb strains resistant to some or all available antibiotics have emerged [5,6], including stains resistant to newly developed antibiotics such as bedaquiline, delamanid, and pretomanid [7,8], highlighting the need for novel treatment strategies for TB. Similarly, antibiotic resistance is of significant concern in infections caused by nontuberculous mycobacteria [9,10].

Mycobacteria subvert the host immune response in a variety of ways. Infection with Mtb attracts macrophages and other innate cells to the site of infection [11] but limits activation and cytokine production of antigen presenting cells [12]. Within macrophages, Mtb, as well as Mm prevent phagolysosomal fusion [13–15], which both precludes activation of macrophages and limits antigen presentation [16–18]. Mtb has also been shown to limit maturation of dendritic cells (DCs) and their migration to lymph nodes [19,20], thereby delaying the onset of adaptive responses [21–23]. Accordingly, in humans adaptive immune responses to Mtb emerge approximately 42 days after exposure [24,25]. Notably mycobacterial infections are accompanied by chronic inflammation, possibly facilitated by secretion of antigenic decoy proteins such as Antigen 85 [26]. Yet the immune response to mycobacteria remains highly effective in most people, and in TB, it is estimated that 90% of those infected with Mtb maintain control of the infection and do not develop active disease [1,27]. However, for all mycobacterial infections, those with immunocompromising conditions remain far more susceptible [9,28]. An important question concerning mycobacteria treatment strategies for people with chronic disease remains how to facilitate a more efficient or efficacious immune response [26].

With a delay in immune responses and chronic inflammation, the infected host forms granulomatous lesions that appear to contain the bacteria and thereby limit its spread. Granuloma formation is in part promoted by cytokines such as tumor necrosis factor [TNF] [29–33] as well as by cell death [34]. Initially, macrophages aggregate and express junctional adhesion proteins that facilitate formation of tight junctions, which enable macrophage to develop properties of epithelial cells, and are recognized histologically as enlarged macrophages with more cytoplasm. These macrophages then surround and ingest infected cells [34,35]. Granulomas also contain neutrophils, dendritic cells, B cells, T cells, and natural killer cells, together with fibroblasts that produce extracellular matrix [ECM] [34]. Epithelioid macrophages and fibroblasts encase the infected cells and limit bacterial dissemination. Many granulomas exhibit necrosis [36,37], which may contribute to chronic inflammation that damages surrounding lung tissue and impairs respiratory function [38]. Moreover, the structure of the granuloma reduces penetrance of antibiotics, resulting in suboptimal antibiotic concentrations within the granuloma, thereby facilitating development of resistance [39,40]. Chronic inflammation results in tissue destruction that contributes to the development of fibrosis and reduces elasticity of lung tissue. As a result, half the patients successfully treated for TB exhibit lasting respiratory impairment [41–43], further exacerbating the economic burden of the disease [44]. While much is known about structure and formation of granulomas, less is known about how to resolve granulomas and restore lung function in TB patients following treatment, or whether resolution would allow bacteria to escape, thereby exacerbating disease.

To address the need for novel therapeutics for mycobacterial disease, we have been developing imatinib as an adjunctive host-directed-therapeutic (HDT). Imatinib is a well-tolerated cancer drug that inhibits tyrosine kinase activity of c-Abl, c-Kit, and platelet derived growth factor receptor [PDGFR], and is the frontline therapy for chronic myelogenous leukemia [CML] and gastrointestinal stromal tumors [GISTs]. As an HDT for infections, imatinib is less likely to engender resistance compared to conventional antimicrobial drugs [45]. Upon infection with Mtb or Mm imatinib induces phagolysosomal fusion in mouse monocytes [46] and human macrophages [47], an effect evident at micromolar concentrations. At low doses in mice, imatinib also induces myelopoiesis [48]. Finally, using the Mm mouse model, in which quantifiable granulomas develop on the tail within two weeks after infection, we showed that prophylactic administration of imatinib at a low inoculum limits development of granulomas [46]. Notably, fibrosis may contribute to bacterial persistence and tissue dysfunction even after discontinuation of antibiotic chemotherapy [38,41,42,49]. Fibrosis in lung, skin and other tissues associated with noninfectious causes is mediated by PDGFR, also a target of imatinib, and imatinib has shown efficacy in these indications [50–55], raising the possibility that the drug may likewise limit fibrosis and granuloma-associated pathology.

The observations that imatinib limits formation of Mm granulomas and induces myelopoiesis, led us to hypothesize that the drug might accelerate the generation of anti-mycobacteria immune responses, and in so doing might also promote resolution of granulomas in a therapeutic context. We show here that imatinib provided after granulomas form limits their growth and/or facilitates their resolution. Analysis of gene expression within tail granulomas indicate that imatinib accelerates appearance of signatures associated with immune cell activation and regulation leading to increased production of many inflammatory cytokines including TNF and IL12P70, as well as the regulatory cytokine IL10 when provided early after infection. However, no changes in cytokine production were evident when the drug was administered later during infection, when granulomas were resolving. Gene ontology [GO] analysis also indicated changes in signatures associated with cell death and survival both early on and when the drug was administered later during infection. To test the involvement of cell death pathways, we found that caspase 8 [CASP8], a key mediator of cell death and survival, mediates imatinib effects *in vitro* and *in vivo*. Thus, our data suggest that imatinib augments immune cell activation and regulation when provided early after infection but does not significantly alter anti-mycobacterial immune responses when provided thereafter. Imatinib also induces cell death and survival responses, which, via CASP8, may facilitate resolution of pathology and tissue damage associated with granulomas.

## Results

### Imatinib limits formation of granulomas in mice infected with Mm

**Fig 1:**
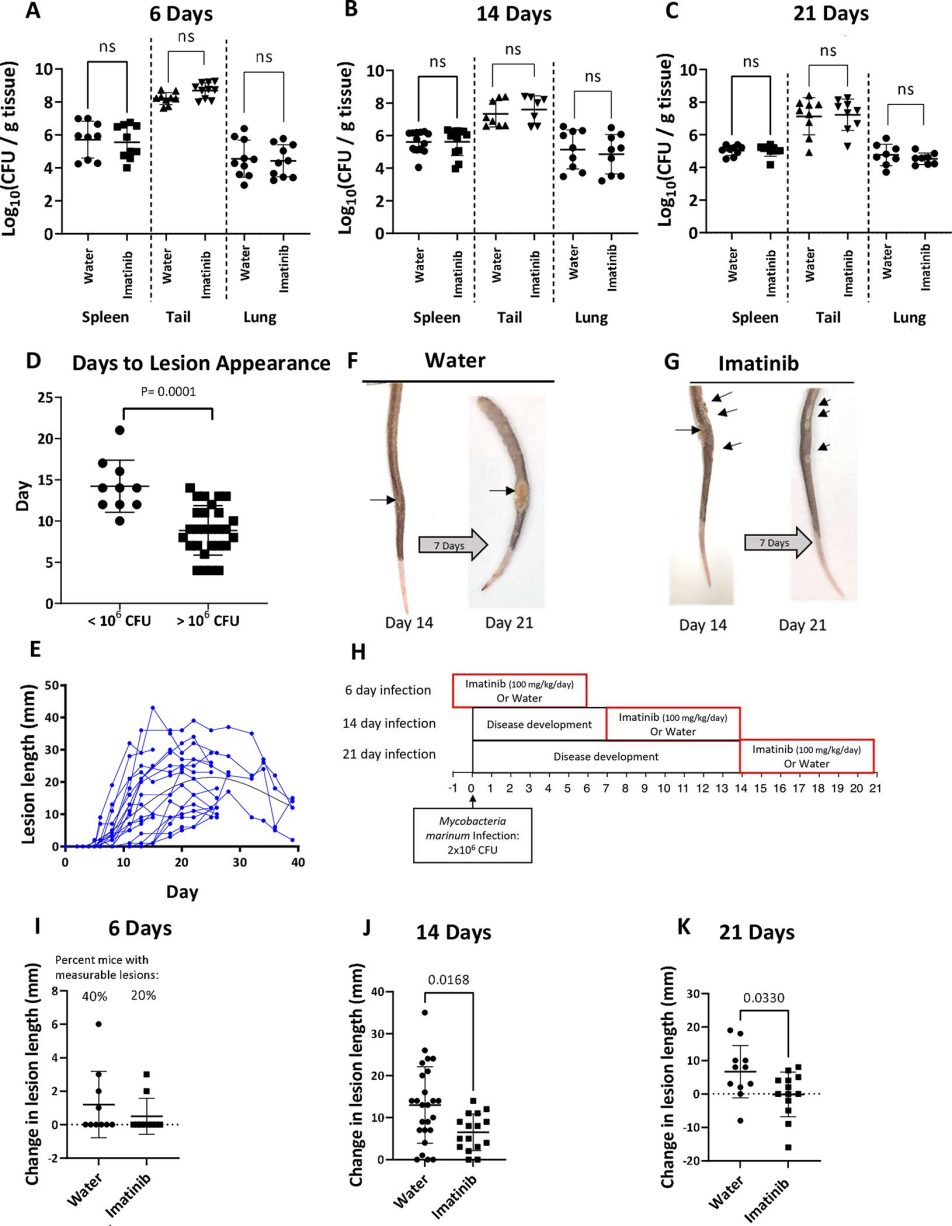
Imatinib causes a reduction in the size of granulomatous lesions in Mm-infected mice. **A-C.** Bacterial load in the spleen, tail, and lung of mice infected with 2x10^6^ CFU of Mm and treated with 100mg/kg/day imatinib via osmotic pump starting day -1, day 7, or day 14 for a total infection period of 6 days **(A)**, 14 days **(B)**, and 21 days **(C)**. **D.** Relationship between the number of days p.i. that lesions are first visible on the tails as a function of the inoculum. High inoculum (>10^6^ CFU/mouse) or a low inoculum (<10^6^ CFU/mouse) of Mm were used (n = 10–28 mice/group). **E.** Time course of lesion size increases and decreases for individual mice following inoculation with 2x10^6^ CFU of *Mm*. Tail lesion size in mm was measured every 1–2 days for each mouse for up to 5.5 weeks p.i.. **F, G.** Representative images of changes in tail lesions from day 14 to day 21 in mice treated with water (**F**) or imatinib (**G**) beginning at day 14. **H.** C57BL/6J mice were infected with 2x10^6^ CFU of *Mm*. Mice were then treated with 100mg/kg/day imatinib or water via osmotic pump starting day -1, day 7, or day 14. Treatment was administered for 7 days before mice were sacrificed. The total infection period was 6 days (n = 10 mice/group), 14 days (n = 16–26 mice/group), or 21 days (n = 11–16 mice/group). **I-K.** Change in lesions size during imatinib or water treatment during infection and treatment protocol shown in **H** for a total infection period of 6 days **(I)**, 14 days **(J)**, or 21 days **(K).** Each data point represents one individual mouse from 2–5 experiments at each timepoint. Statistical tests used for comparisons in **A-D** and **I-K** was a two-tailed Mann-Whitney U test, with p values indicated. A p value > 0.05 was considered not significant (ns).The mean +/- SD, for each group is presented to show the variance in the data.

In previous work, imatinib mesylate, administered to mice starting one day prior to IV infection for one week at an inoculum of ~10^5^ CFU Mm/ mouse, reduced CFUs in the spleen or lung, whereas no reductions were evident in water treated control animals [46]. However, at inoculums >10^6^ CFU Mm/mouse, the drug was without effect on CFUs in any tissue at 6-, 14-, or 21-days post infection [p.i.] (Fig 1A–1C). Notably, in contrast to previous work, an inoculum of 2x10^6^ CFU Mm/mouse was used here because, compared to an inoculum of <10^5^ CFU Mm/mouse, it induces more rapid formation of granulomatous lesions on the tail, usually within 4–12 days (Fig 1D), which continue to increase in size before reaching their maximal extent by 14–21 days p.i. (Fig 1E). Lesion size was quantified over time by measuring the length of each lesion on the surface of the tail and computing the change in lesion size over the treatment period (Fig 1F and 1G). With imatinib treatment beginning one day prior to infection with 10^7^CFU/mouse, development of granulomatous lesions was limited during the first 6 day of infection as previously reported [46]. In an experiment using an inoculum of 2x10^6^ CFU Mm/mouse (Fig 1H), only 20% of imatinib-treated animals developed lesions during the first week compared to 40% of animals treated with water (Fig 1I). To determine whether imatinib limited development of actively growing lesions, the drug was administered starting at day seven p.i. and continuing until day 14 (Fig 1H). Over this time period, lesions from water-treated mice grew by an average of 13 mm, whereas lesions from imatinib-treated animals grew by an average of 6.5mm (Fig 1J, 1F and 1G). To determine the effects of imatinib on established granulomas, lesions were allowed to develop for 14 days prior to administration of imatinib, and then for an additional seven days with imatinib present (Fig 1H). Whereas lesions on control mice grew by an average of 6.6mm during this period, no net growth was evident in mice treated with imatinib, with lesion size increasing in some animals (6 of 13 animals), but either not changing (3 of 13 animals) or decreasing (4 of 13 animals) in others (Fig 1K). Together, these data suggest that imatinib limits formation and growth of granulomas in a manner that does not depend on bacterial load.

**Fig 2:**
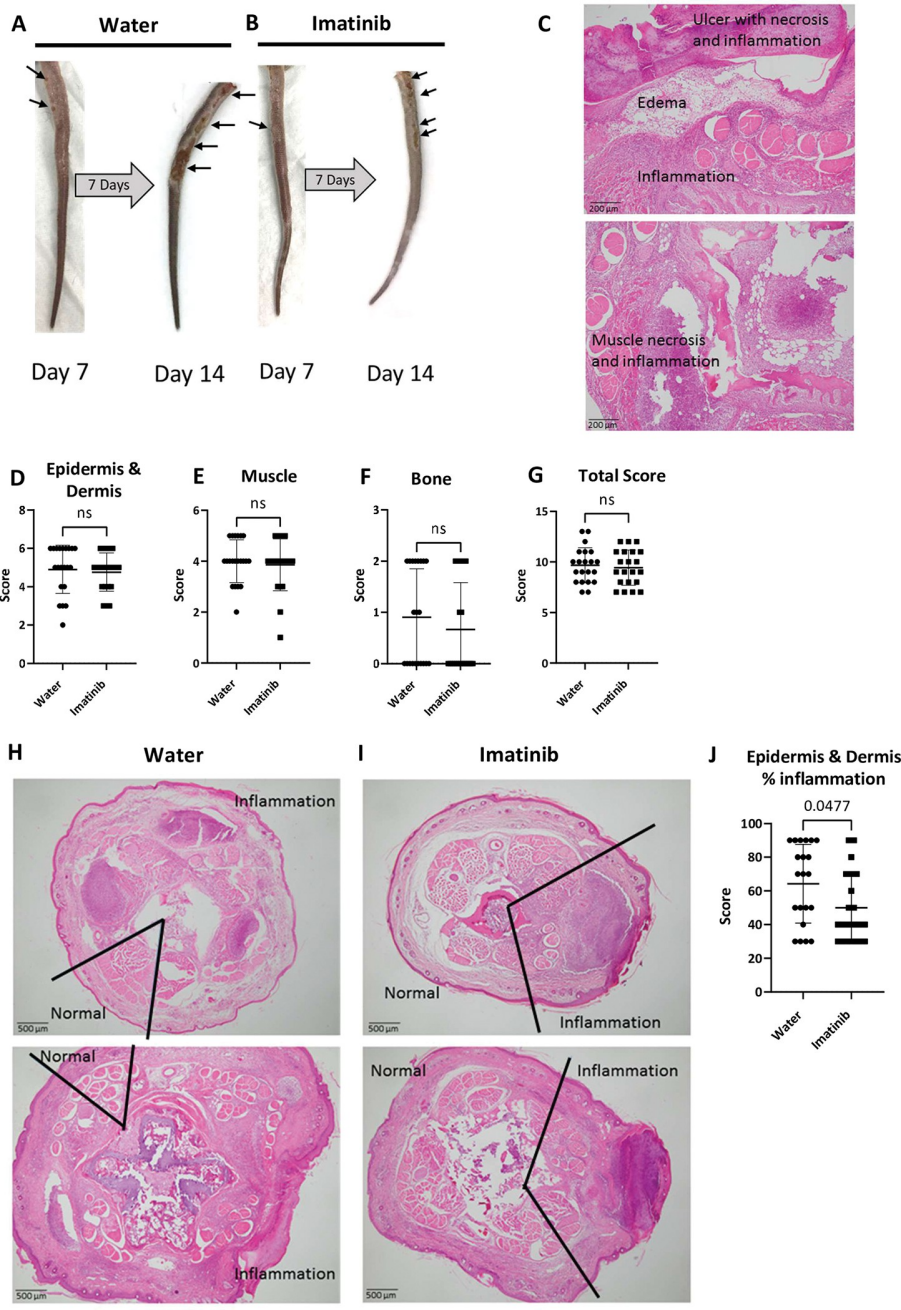
Imatinib reduces area of inflammation. **A, B** Representative images of tail lesions from the same animal at day 7 and day 14. Mice were treated with water (**A**) or imatinib at 100mg/kg/day (**B**) beginning at day 7. **C.** Following sacrifice on day 14, cross sections of the tail were cut and stained with H&E. Representative images of lesions show ulceration, epidermal necrosis, edema, dermal inflammation, muscle necrosis, and muscle inflammation, in the tail sections. Magnification is 100x. **D.** Composite pathology scores for the epidermis and dermis accounting for ulceration, necrosis, calcification, edema, acute inflammation, and chronic inflammation. **E.** Composite pathology scores for the muscle accounting for necrosis, edema, thrombi, acute inflammation, and chronic inflammation. **F.** Composite pathology scores for the bone accounting for necrosis and inflammation. **G.** Total composite pathology scores based on scores from epidermis and dermis, muscle, and bone. **H, I.** Representative images of whole tail sections at 14 days post infection from mice treated with water **(H)** or imatinib at 100 mg/kg/day **(I)** demonstrating percent of the tissue section showing inflammation. Magnification is 40X. **J.** Quantification of the area of the tissue sections showing inflammation. Scoring in **D-G** and **J** is based on mice from 4 separate experiments (n = 21 animals in each treatment group). Comparisons analyzed by a two tailed Mann-Whitney U test with p values indicated and p values > 0.05 considered not significant (ns). The mean +/- SD, for each group was graphed to show the variance in the data.

We next examined histopathological features within the tail lesions using hematoxylin and eosin (H&E) stained tail sections taken at day 14 with or without imatinib treatment for 7 days (Fig 2A and 2B). Pathology scoring of the sections was blinded to treatment received. Scores were assigned for the number of ulcers, inflammation, and necrosis in the epidermis and dermis, or muscle, or bone (Figs 2C and S1A–S1N). We did not observe significant differences between the two groups with respect to the presence of inflammation and necrosis in epidermis and dermis (Fig 2D), muscle (Fig 2E) or bone (Fig 2F) or with total pathology scores (Fig 2G). However, the percentage of tissue displaying inflammation was significantly reduced in the mice receiving imatinib (Fig 2H–2J). Acid fast staining organisms remained evident within necrotic regions of lesions regardless of treatment (S1O Fig). Together, these data suggest imatinib does not induce changes in microscopic composition of granulomatous lesions but does reduce the area of inflammation.

### Imatinib upregulates immune genes and downregulates ECM genes in uninfected animals

**Fig 3:**
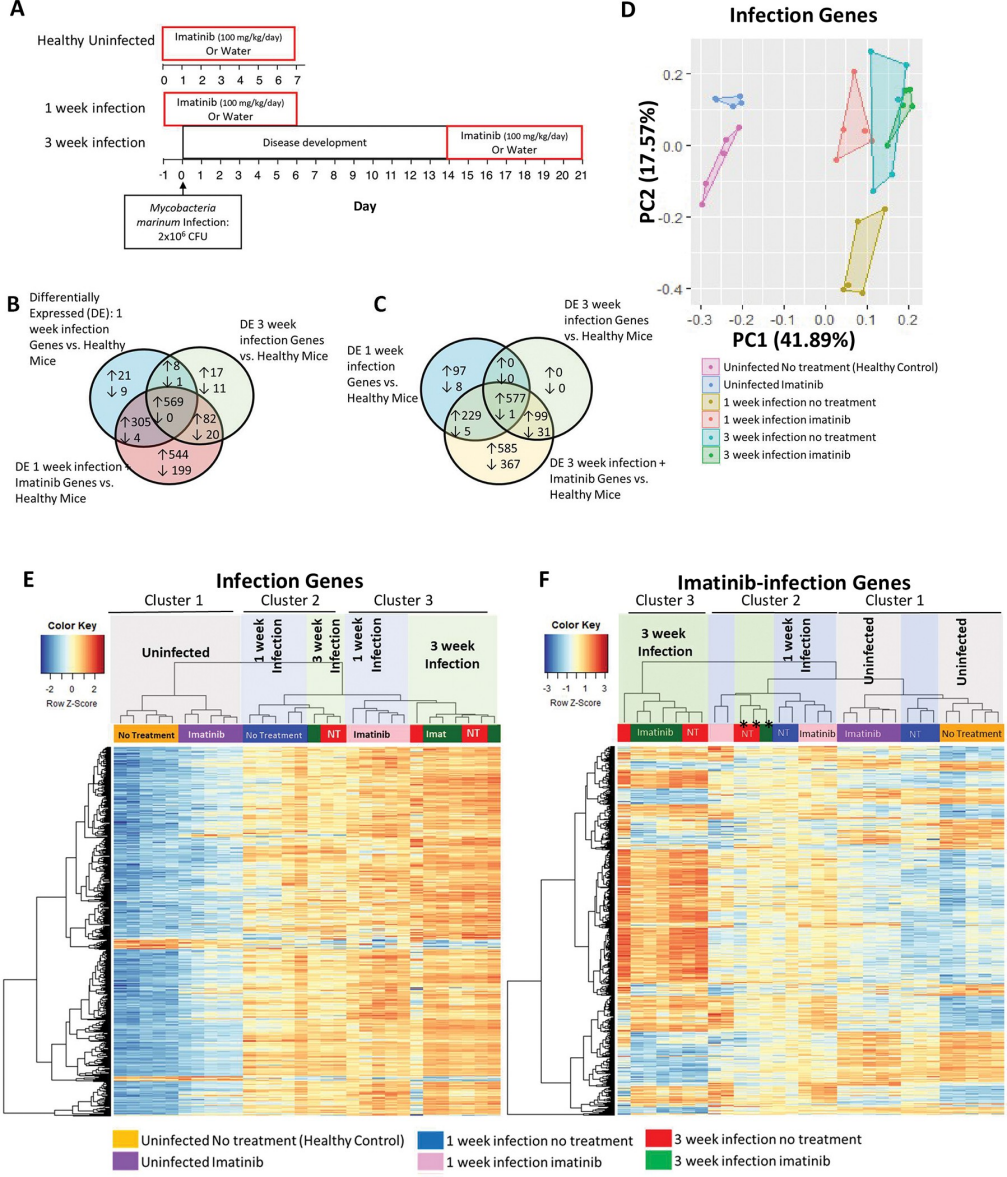
Imatinib upregulates a similar but larger number of genes with infection and at an earlier time point and with greater reliability than infection alone. **A.** Schema of administration of imatinib or water to C57BL/6J mice left uninfected or infected with 2x10^6^ CFU of *Mm*. Mice were treated with imatinib (100mg/kg/day) or water for seven days starting day -1 or day 14 in infected group, or starting at day 0 in uninfected group, and then sacrificed seven days later (n = 5 mice/group). **B. “**Infection genes” were identified by determining differentially expressed genes between the uninfected water-treated group and the one week infection water-treated group (917 genes, False discovery rate [FDR]< 0.05; blue circle) or the three week infection water-treated group (708 genes, FDR < 0.05; green circle) for a total of 1047 unique “infection genes” (578 genes were present at both time points). Genes induced by imatinib at 1 week infection were identified by determining differentially expressed genes between the uninfected water treated group and 1 week infection plus imatinib group (1723 genes, FDR < 0.05; red circle). **C.** Genes induced by imatinib at 3 weeks p.i. were identified by determining differentially expressed genes between the uninfected water-treated group to three week infection plus imatinib group (1894 genes, FDR < 0.05; yellow circle). This group was then compared to Infection genes at 1 week (blue circle) and 3 weeks (green circle) previously defined in **B. D.** The top two principal components for the variance of expression levels in all 6 of our treatment/infection groups of the 1047 “Infection Genes” identified in **B** are presented. **E.** Infection genes identified in **B** were graphed in a heat map with unsupervised hierarchical clustering. **F.** Genes specific to imatinib treatment at both time points identified in **B** and **C,** defined as “Imatinib-infection Genes,” were graphed in a heat map with unsupervised hierarchical clustering.

To determine how imatinib affects gene expressions profiles within granulomas, RNA-seq was performed on tail sections in close proximity to the mock injection site from uninfected animals, or, alternatively, on areas of the tail with visible lesions from mice infected for one or three weeks with Mm (accession no. **GSE215176**). Infected groups included mice treated with water or imatinib beginning one day prior to Mm infection for 7 days [“one-week infection” group (Fig 3A)] and mice in which lesions were allowed to develop for 14 days and then treated with water or imatinib for an additional 7 days [“three-week infection” group (Fig 3A)]. RNA-seq profiles from tails of uninfected mice treated with water or imatinib served as a baseline with which to determine differentially expressed genes associated with infection based on a false discovery rate [FDR] of 0.05.

In the absence of infection, imatinib differentially regulated 514 genes compared to water controls, of which 373 were upregulated and 141 were downregulated. GO analysis of upregulated genes indicated that imatinib regulated diverse cellular processes, many of which were associated with the function, activation, or regulation of the immune response (S2A Fig). Imatinib has been reported to induce myelopoiesis in mice at the dose used [48]; accordingly, GO terms associated with neutrophil function (granulocyte activation and migration) and myeloid cell differentiation were upregulated with imatinib. GO processes significantly downregulated with imatinib include skin-epidermis development and extracellular matrix [ECM] organization, which was ascribed to a significant reduction of 17 collagen subunits including *col3a1*, c*ol1a2*, *col1a1*, *col5a1*, and *col8a2* (S2B Fig). Thus, without infection, imatinib upregulated genes and processes associated with immune function, and downregulated genes associated with ECM.

### Imatinib rapidly induces expression of infection genes

Genes regulated by infection (“infection genes”) were identified as those differentially expressed at the one- or three-week timepoints following infection as compared to expression levels in uninfected mice treated with water (controls). We identified 903 genes upregulated at the one-week time-point, of which 577 remained upregulated at three weeks (Fig 3B and 3C). GO analysis of the infection genes upregulated at one week indicated processes involved in immune responses and cytokine production, including responses to interferon-gamma and positive regulation of TNF, IL6, and IL1β production (S2C Fig). At the three-week time point, 99 additional upregulated genes were identified as infection genes, for a total of 676 genes at this time point. GO analysis of upregulated infection genes at three weeks indicated immune-related processes, including regulation of the cytokines IL6, IL1, IL12, IL10, IL8 and TNF, as well as macrophage activation, and responses to wounding (S2D Fig). GO analysis of genes downregulated by infection at either the one- or three-week timepoints did not identify any processes.

We next examined how imatinib impacted expression of infection genes. To do this, the variance of infection genes without or with imatinib was analyzed by principal component analysis ([PCA]; Fig 3D). Uninfected mice clustered together in terms of PC1 (pink box), and imatinib treatment for one week shifted the variance, though only in PC2 (compare pink and blue boxes; Fig 3D). Infection alone for one week shifted the variance primarily along the PC1 axis (gold box; Fig 3D), and imatinib treatment again shifted the variance only in PC2 (compare gold and red boxes; Fig 3D). Infection for three weeks shifted the variance in PC2 (compare gold and teal boxes; Fig 3D) to a position near that seen with imatinib treatment at one week (compare red and teal boxes; Fig 3D). Imatinib treatment at this time point produced little additional shift (compare green and teal boxes), however, less variance in expression was apparent, as indicated by the tighter clustering of the data points. Overall, imatinib altered the pattern of variance in infection genes such that the variance at the one-week timepoint with imatinib resembled that seen at three weeks without imatinib, and at three weeks, imatinib further restricted the variance.

A similar pattern was evident when expression levels for the infection genes were displayed on a heatmap with unsupervised hierarchical clustering (Fig 3E). Three main clusters were evident. Uninfected mice displayed low expression levels for most infection genes, and imatinib induced a marginal increase in expression of some of these genes (Cluster I). A second cluster included the one-week infection water-treated group together with a few mice from the three-week infection timepoint, which displayed a low bacterial burden (Cluster 2). A third cluster included all the one-week infection imatinib-treated mice, together with the remaining three-week infection mice (Cluster 3). As in the PCA plot, four of the five imatinib-treated mice in the three-week infection group displayed similar gene expression profiles, whereas expression levels of infection genes in water-treated mice exhibited more variance between animals. Taken together, these data indicate that treatment with imatinib induced a pattern of gene expression at one week of infection that resembled that seen at three weeks, and imatinib at three weeks of infection resulted in more uniform expression of infection genes.

### Imatinib augments a gene signature associated with infection

We next identified genes differentially regulated by imatinib. When directly comparing differences in gene expression with infection and with or without imatinib at the one- and three-week timepoints, no genes were identified as differentially expressed with imatinib at either timepoint, as defined by an FDR of less than 0.05. Thus, treatment with imatinib did not cause significant changes in genes expressed during infection. We next compared genes expressed with imatinib and infection at the one- and three-week timepoints to that of water-treated uninfected controls [called imatinib genes]. At the one-week infection timepoint, imatinib upregulated 1,418 genes compared to 903 infection genes in infected mice treated with water alone (compare blue and red circles in Fig 3B). Of the 1,418 genes, 874 were shared amongst the infection and infection plus imatinib groups, and 569 were core infection genes that were upregulated at both one and three weeks of infection (Fig 3B). At the three week timepoint, imatinib upregulated 1,490 genes, including all 676 three week infection genes upregulated with water, as well as 229 additional genes also upregulated at the one week time point (Fig 3C). Thus, at each timepoint, most infection genes are induced by imatinib, together with an additional 544 genes at one week and 585 genes at three weeks. Of the imatinib-induced additional genes, ~300 genes are specific to each timepoint, and 227 genes are upregulated by imatinib at both timepoints (S2E Fig). Thus, imatinib induced 902 additional genes in infected animals, not originally identified as infection genes (“imatinib-infection genes”).

When expression of the imatinib-infection genes was displayed in a heat map with unsupervised hierarchical clustering, three clusters were evident (Fig 3F). Cluster 1 contained all the uninfected mice together with 3 of 5 one-week infection mice with no treatment. The second cluster (Cluster 2) contained the rest of the mice infected for one week, including all the one week infection mice treated with imatinib, and a few of the three week infection mice that had lower bacterial CFUs in the spleen and tail (*, Fig 3F). The third cluster contained all remaining three week infection mice, including those treated with imatinib or water. Taken together, these data indicate that imatinib treatment augments the infection gene signature to include additional imatinib-infection genes at both the one week and three week time points, which are similarly regulated without imatinib treatment, but not as reliably expressed in water-treated animals.

### GO analysis of imatinib-infection genes reveals signatures associated with immune activation and regulation, and with cell death

We surmised that the imatinib-infection gene signature might correlate with observed changes in granuloma formation or resolution. To test this possibility, we further characterized the imatinib-infection gene signature by GO analysis, focusing first on the one week timepoint, where gene expression differences between the imatinib- and water-treated animals were most apparent and granulomas were just beginning to form, and then at the three week time point where imatinib treatment initiated later in infection reduced both granuloma size and inflammation.

**Fig 4:**
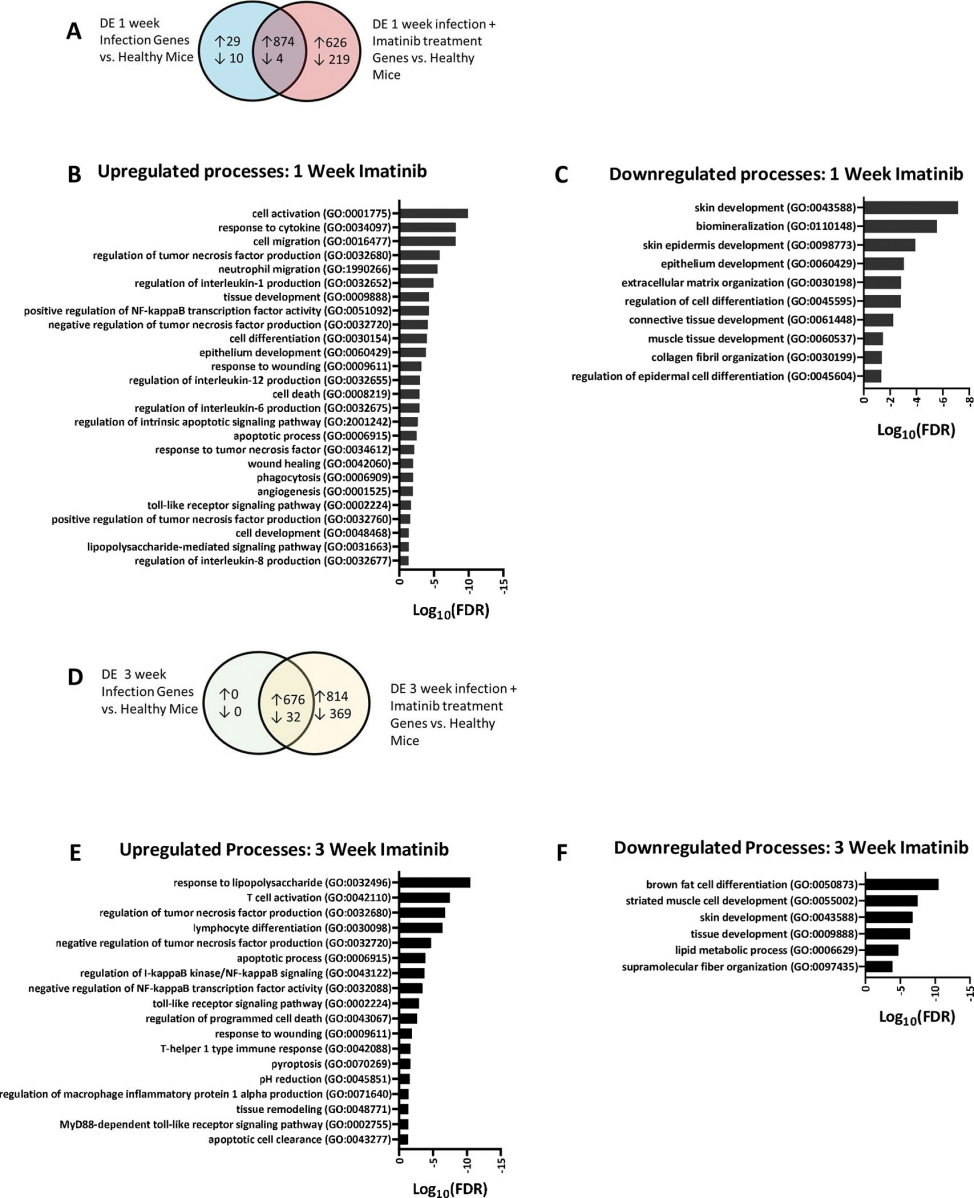
GO analysis of imatinib-infection genes. **A.** Comparison of 1 week infection related genes and 1 week infection plus imatinib genes as described in Fig 3B**. B.** Selection of GO terms identified by analysis of 626 upregulated genes from **A** unique to imatinib treatment at 1 week infection. **C.** Selection of GO terms identified by analysis of 219 downregulated genes from **A** unique to imatinib treatment at 1 week infection. **D-F.** Comparison of three week infection genes identified in Fig 3B, to 3 week infection with imatinib genes identified in Fig 3C **(D)**. Selection of GO terms identified by GO analysis of the 814 upregulated genes **(E)**, and the 369 downregulated genes **(F)** specific to imatinib treatment at the 3 week infection time point.

At the one week timepoint, imatinib upregulated 626 more genes than infection alone and downregulated 219 genes (Fig 4A). GO analysis of the upregulated genes indicated many immune system processes, including cell activation, response to and regulation of cytokines (IL1, IL12, IL6, TNF, IL8), wound healing, and cell death (Fig 4B). Notably, processes downregulated by imatinib during infection (Fig 4C) were similarly downregulated by imatinib in uninfected mice (S2B Fig). These included skin development as well as ECM and collagen organization (Fig 4C).

At the three week infection timepoint, imatinib upregulated 814 more genes than infection alone and downregulated 369 genes (Fig 4D). GO analysis indicated processes associated with upregulated genes at three weeks were similar to those upregulated by imatinib at one week. These include responses to lipopolysaccharide, cytokine regulation, and cell death (Fig 4E). Downregulated genes at the three week timepoint were associated with lipid metabolism, tissue development, and supramolecular fiber organization (Fig 4F). Thus, processes up- or down-regulated with imatinib at three weeks were similar to those evident at one week.

### Imatinib enhances production of inflammatory cytokines only during initial stages of infection

**Fig 5:**
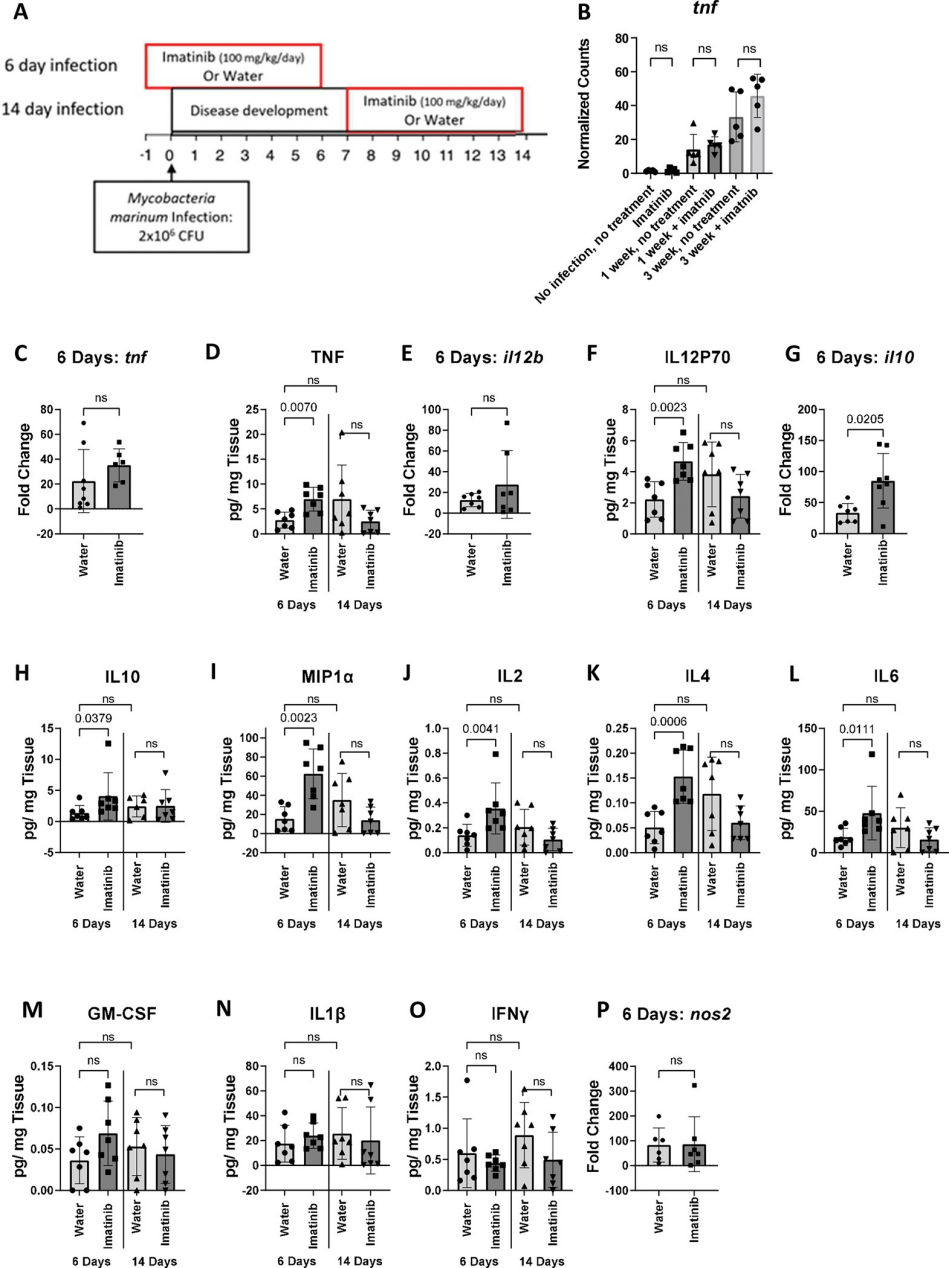
Imatinib treatment increases cytokine production in tissue at 6 day post infection but not 14 days. **A.** C57BL/6J mice were infected with 2x10^6^ CFU of *Mm*, for a total of 6 or 14 days. Mice were either treated with imatinib (100mg/kg/day) or water starting day -1 or 7 relative to the infection start day for a total of 7 days before mice were sacrificed at day 6 or 14 (n = 6–12 mice/group). **B.** Normalized gene counts from RNAseq experiment (described in Fig 3A) mapped to the *tnf* gene. **C-P.** RNA and protein were isolated from sections of the mouse tail at 6 or 14 days p.i with water or imatinib treatment (100 mg/kg/day). qPCR was used to measure mRNA levels of *tnf* **(C),** *il12b* **(E),** *il10* **(G),** and *nos2***(P)** (n = 7 mice/group) at the 6 day time point. Cytokine and chemokine content was measured via a multiplexed chemiluminescent assay for TNF **(D)**, Il12p70 **(F)**, Il10 **(H),** MIP1α **(I)**, IL2 **(J)**, IL4 **(K)**, IL6 **(L)**, GM-CSF **(M)**, IL1β **(N)**, and IFNγ **(O)** on the protein isolated from the mouse tails at the 6 and 14 day timepoints (n = 7 mice/group). Statistical test used in **B-P** was a two-tailed Mann-Whitney U test, with p values indicated. p values > 0.05 were considered not significant (ns). The mean +/- SD, for each group is presented to show the variance in the data.

To corroborate the GO analysis, we next quantified cytokine expression and protein levels in granulomas using imatinib administration beginning at day -1 through day 6 or beginning at day 7 and extending through day 14 (Fig 5A). In the RNAseq analysis, infection increased levels of *tnf* at the one week timepoint, an effect slightly augmented by imatinib, and levels increased further by three weeks, though these differences did not reach statistical significance at the p<0.05 level (Fig 5B). Levels of *tnf* RNA by qPCR were likewise not significantly different with imatinib plus infection compared to infection alone at day 6 (Fig 5C). However, at this time point, levels of TNF protein were significantly higher with imatinib plus infection compared to infection alone (Fig 5D). The levels of *il12b* RNA, which encodes a subunit of IL12p70, were similar in both water and imatinib groups at day 6 (Fig 5E). However, protein levels of IL12p70 were strongly upregulated by imatinib at day 6 p.i. (Fig 5F). Levels of *il10*, which encodes the immune regulatory cytokine IL-10, significantly increased with infection plus imatinib at day 6 (Fig 5G) compared to infection alone, an effect recapitulated in measurements of IL10 protein levels (Fig 5H). Various other cytokines and chemokines were observed to be present at higher concentrations with imatinib at day 6 p.i., including MIP1α (Fig 5I), IL2 (Fig 5J), IL4 (Fig 5K), and IL6 (Fig 5L). Levels of other cytokines including GM-CSF (Fig 5M), IL1β (Fig 5N), and IFNγ (Fig 5O), were not changed with imatinib at this time point. Likewise, *nos2*, which is upregulated in Mtb granulomas and associated with disease protection, remained unchanged with imatinib at day 6 (Fig 5P). Together, these data indicate that at the day 6 timepoint, imatinib induces both pro-inflammatory cytokines as well as IL10, a regulatory cytokine that limits inflammation.

We next measured cytokine and chemokine mRNA and protein levels in the granulomas of mice receiving imatinib from day 7 to day 14 p.i. (Fig 4A). Notably, none of the cytokines and chemokines assessed were evident at higher levels with this treatment regimen (Fig 5D, 5F and 5H–5O). Indeed, levels of TNF (Fig 5D), IL12P70 (Fig 5F), MIP1α (Fig 5I), IL2 (Fig 5J), IL4 (Fig 5K), IL6 (Fig 5L), and IFNγ (Fig 5O) were slightly lower than the water control, though this difference did not meet the threshold for statistical significance. Levels of IL10 (Fig 5H), GM-CSF (Fig 5M), and IL1β (Fig 5N) were all similar between the water and the imatinib groups. These data, in conjunction with the cytokine and chemokine measurements at day 6, suggest that while imatinib induces markers of immune activation and regulation when provided during initial phase of infection when granulomas are initially forming, such induction was not evident when the drug was provided at later stages of infection, when granulomas are continuing to enlarge.

### Imatinib treatment of BMDMs increases initial cytokine production

**Fig 6:**
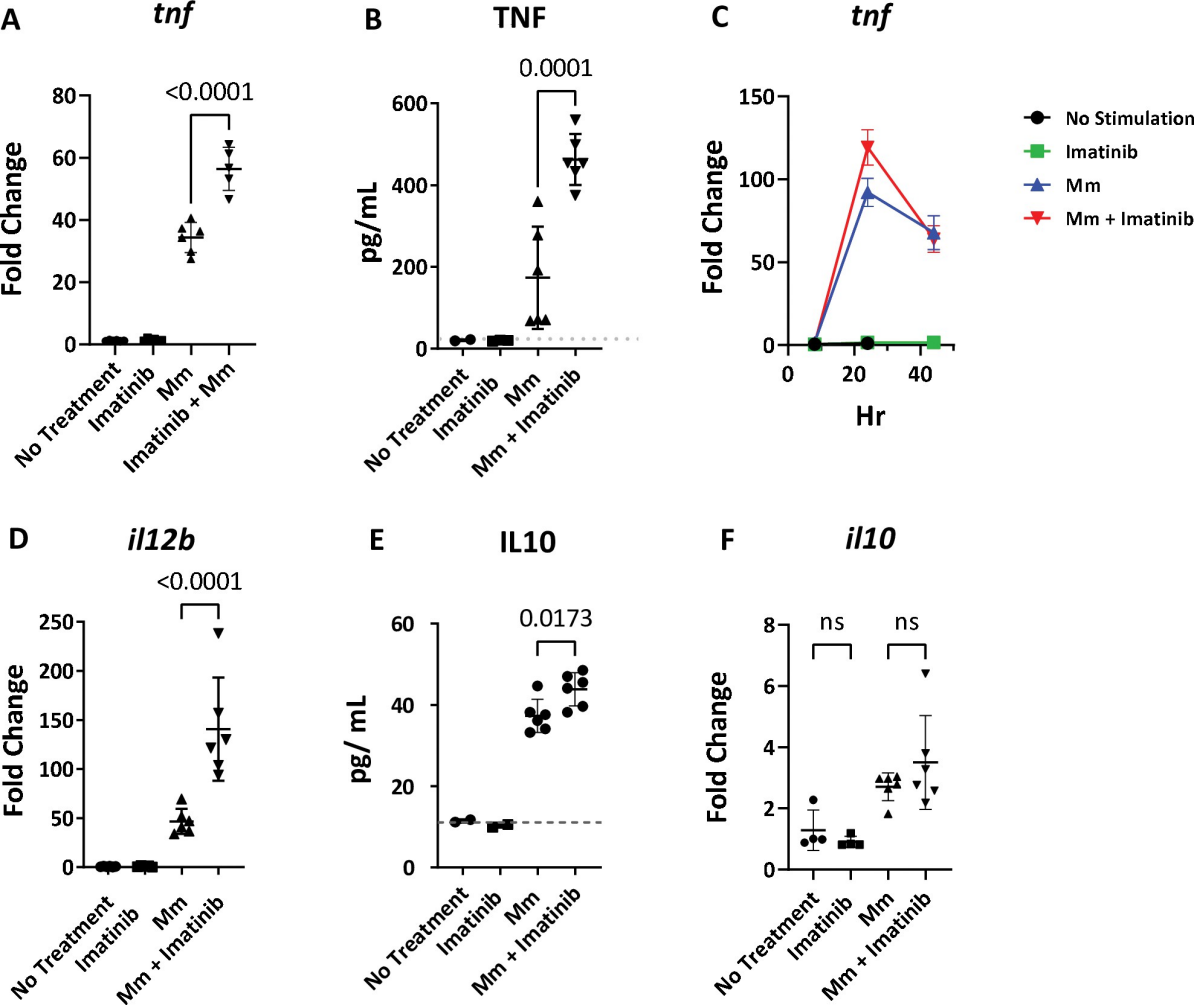
Imatinib treatment increases cytokine production in BMDMs. **A-F.** BMDMs derived from C57BL/6J mice infected with Mm at an MOI of 10 for 8, 24 or 44 hrs at 37°C. RNA was isolated from the cells and qPCR was used to measure mRNA levels of *tnf* **(A),** *il12b* **(D),** and *il10* **(F)** at 24 hrs (n = 5-6wells/ group). qPCR was used to measure levels of *tnf* in the cells 8,24, and 44hrs (**C;** n = 2 wells/ group; data are representative of 2 separate experiments). ELISAs were used to measure TNF **(B)**, and Il10 **(E)** in the media at 24 hrs (n = 2–6 wells/group). Statistical test used in was one-way ANOVA, with p values indicated. A p values > 0.05 was considered not significant (ns). The mean +/- SD, for each group was graphed to show variance of data.

Macrophages are among the first cells infected by mycobacteria and a source of cytokines such as, TNF, IL12P70, and IL10 [56,57]. We next assessed whether imatinib induced cytokine changes in cultured bone marrow derived macrophages [BMDM] from C57BL/6J mice. Unstimulated BMDMs were infected with Mm at an MOI of 10, exposed to imatinib, and mRNA levels in the cells or protein levels in the media assessed 8, 24, and 44 hours later. Compared to untreated cells, imatinib induced expression of *tnf* mRNA (Fig 6A) and enhanced release of TNF into the media (Fig 6B) compared to infection alone, when measured at 24 hours p.i. Notably, *tnf* mRNA levels evident with imatinib returned to a baseline level similar to that of control infected cells by 44 hours p.i. (Fig 6C). Il12p70 protein was not produced in measurable levels by BMDMs following Mm infection though levels of *il12b* mRNA were increased with imatinib treatment (Fig 6D). Imatinib also induced higher levels of secreted IL10 (Fig 6E), though, in contrast to *in vivo* results, *il10* mRNA levels remained unchanged (Fig 6F). Levels of *nos2* mRNA were strongly induced with infection over the first 24 hours and continued to increase thereafter, and imatinib limited such increases (S3A and S3B Fig). Despite imatinib reducing levels of *nos2*, a marker of M1 macrophage activation, levels of the M2 macrophage activation markers *chil3l3* (YM1) and *arg1* were unchanged (S3C and S3D Fig). These data indicate that in infected BMDMs, imatinib augments production of TNF, a marker of macrophage activation, and IL10, mirroring effects seen *in vivo* (Fig 5D and 5H), though *in vivo* regulation by imatinib of other factors, such as *nos2* was not recapitulated in these cells.

### Imatinib reduces necrosis and increases cellular survival in BMDMs upon Mm infection

Since cytokine levels are only altered with imatinib early during infection, effects on cytokine levels *per se* cannot account for changes to pathology evident with imatinib at later timepoints. Various cytokines, including TNF, which is induced by imatinib (Figs 5D and 6B), regulate cell death and survival pathways [58–60], and have been implicated in host control of Mtb infections and granulomas in humans [29–32]. Notably, GO signatures associated with cell death are induced by imatinib at both early and later stages of Mm infection (Fig 4).

**Fig 7:**
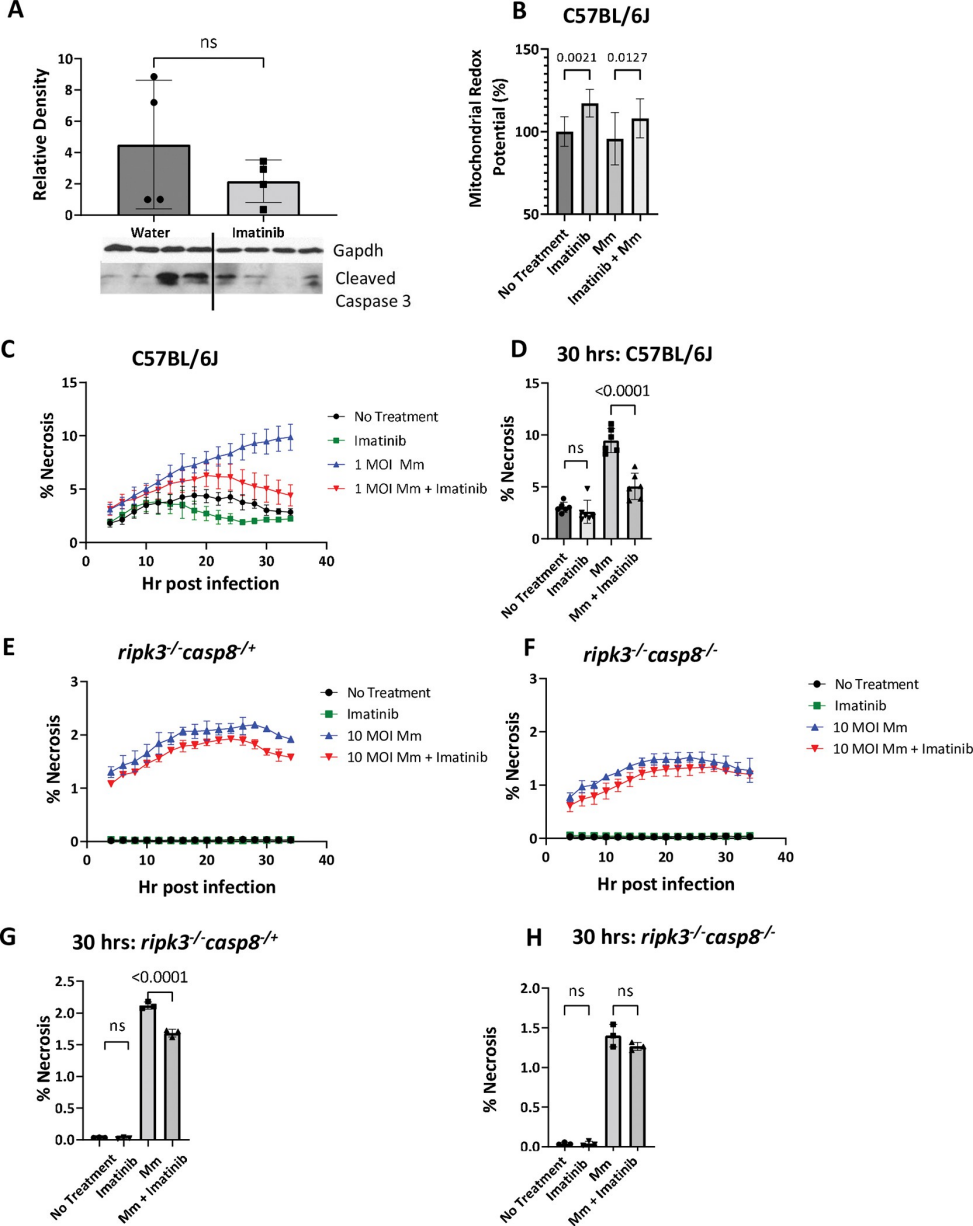
**Imatinib limits necrosis in BMDMs, which depends upon caspase 8 [CASP8]. A.** BMDMs derived from C57BL/6J mice were infected with Mm at an MOI of 5 and left untreated or treated with 1μM imatinib. Cell viability was assessed 20 hrs. p.i. by measuring fluoresecence of resorufin, and indicator of redox potential. All groups were normalized to the average of the no infection/no treatment group (n = 10 wells/group; data shown are representative of 3 separate experiments). **B.** Western analysis of cleaved caspase 3 was performed on protein isolated from sections of the mouse tail at 6 days p.i. with water or imatinib treatment (100 mg/kg/day) for 7 days**. C,D.** BMDMs derived from C57BL/6J mice were infected with Mm at an MOI of 1 and left untreated or treated with 1μM imatinib. Cells were imaged every 2 hrs with Cytotox dye (n = 6 wells/group, representative of 3 separate experiments), an indicator of necrosis. Percent (%) necrosis was quantified at each time point and graphed as a function of time post infection (**C**). Statistical analysis was performed on data at 30 hrs post infection and graphed in **D**. **E-H.** BMDMs derived from *ripk3*^*-/-*^ *casp8*^*-/*+^ or *ripk3*^*-/-*^ *casp8*^*-/*-^ mice were infected with Mm at an MOI of 10 and left untreated or treated with 1μM imatinib. Cells were imaged every 2 hrs with Cytotox dye (n = 3 wells/ group, representative of 3 separate experiments). Data is show as a function of time **(E,F)**, and at the 30 hr timepoint **(G,H)** for the *ripk3*^*-/-*^ *casp8*^*-/*+^ or *ripk3*^*-/-*^ *casp8*^*-/*-^ BMDMs, respectively. Statistical test used in **A, D, G, & H** was one-way ANOVA. Statistical test used in **B** was two-tailed Mann-Whitney U test, with p values indicated. A p value > 0.05 considered not significant (ns). The mean +/- SD, for each group was graphed to show variance of data.

To determine whether imatinib regulates cell death in granulomas, the level of cleaved caspase 3, an indicator of apoptosis, was quantified by western analysis in the tails of the mice infected with Mm and treated for 7 days with water or imatinib. Cleavage of caspase 3 was evident with infection, with some animals showing higher levels than others; however, on average, imatinib treatment did not affect levels of cleaved caspase 3 (Fig 7A), nor, by inference, the level of apoptosis. Necrosis was evident in most tissue sections at day 14 p.i. (S1C, S1H and S1M Fig), however, no quantifiable differences were apparent with imatinib as tissue sections collected contained visible granulomas, which often contain necrotic areas, thus skewing our sample to include necrotic regions in both groups.

To determine whether imatinib directly affects cell viability, mitochondrial redox potential was assessed in C57BL/6J BMDMs. To do this, cells were treated with imatinib with or without infection with Mm at an MOI of 5, and mitochondrial redox potential measured 24 hours later. Imatinib treatment of uninfected BMDMs increased redox potential by ~15%, and no changes were evident upon infection with Mm compared to uninfected cells. However, imatinib treatment increased redox potential by 8% in infected cells (Fig 7B), indicating a survival benefit with the drug.

To determine if this imatinib-induced survival benefit protects the cells against necrotic cell death, C57BL/6J BMDMs were cultured with or without imatinib and infected with Mm at an MOI of 1 or left uninfected. Cells were imaged over a 36-hour period in the presence of Cytotox reporter, which labels cells that have lost membrane integrity, an indication of necrosis. With or without imatinib, some necrotic cell death was evident after four hours p.i., which plateaued after 24 hours. Imatinib reduced the amount of necrosis between 16 and 24 hours (compare black and green lines; Fig 7C). Upon infection with Mm at an MOI of 1, the percentage of cells undergoing necrosis increased over time (blue line, Fig 7C). With imatinib, the levels of necrosis in infected cells initially increased over 20 hours at a rate similar to that in untreated cells. After 24 hours, levels of necrosis in untreated cells continued to increase whereas necrosis in imatinib-treated cells declined to levels seen in uninfected cells (compare red and blue lines; Fig 7C). By 30 hours p.i., the level of necrosis with infection was 2-fold lower in imatinib-treated cells compared to controls (Fig 7D). Taken together, these data indicate that imatinib increases survival of BMDMs and limits necrosis.

### Imatinib effects on necrotic cell death in BMDMs depend upon caspase 8 [CASP8]

To test the possibility that imatinib might regulate cell death or survival, imatinib effects were assessed in BMDMs derived from mice with deficiencies in CASP8, a key mediator of cell death and survival [61–63]. Cells from mice lacking receptor-interacting protein kinase 3 (RIPK3), an important mediator of TNF-induced necroptosis [64], but heterozygous for *casp8* (*ripk3*^*-/-*^ *casp8*^*-/+*^) do not undergo necroptosis, whereas cells from mice lacking both *ripk3* and *casp8* (*ripk3*^*-/-*^ *casp8*^*-/-*^) can neither undergo necroptosis nor extrinsic apoptosis [65,66]. Without infection, little necrotic cell death was evident in BMDMs derived from either *ripk3*^*-/-*^ *casp8*^*-/+*^mice or *ripk3*^*-/-*^ *casp8*^*-/-*^ mice, and no additional effect of imatinib was discernable (Fig 7E and 7F; Black and green lines). Induction of discernable levels of cell death in BMDMs from *ripk3*^*-/-*^ *casp8*^*-/+*^ or *ripk3*^*-/-*^ *casp8*^*-/-*^ required infection with Mm at an MOI of 10 rather than an MOI of 1 used in C57BL/6J BMDMs, though even at this MOI, the percentage of cells undergoing necrosis was still lower than in C57BL/6J cells (compare Fig 7C, 7E and 7F). With infection, BMDMs from *ripk3*^*-/-*^ *casp8*^*-/+*^ and *ripk3*^*-/-*^ *casp8*^*-/-*^ showed similar initial levels of necrosis without imatinib, which increased over 24hrs before plateauing (Fig 7E and 7F). With imatinib treatment, *ripk3*^*-/-*^ *casp8*^*-/+*^ showed statistically significant reductions in the amount of necrosis by 30 hrs. p.i. (Fig 7E and 7G; compare blue and red lines). However, imatinib did not affect the level of necrosis in *ripk3*^*-/-*^ *casp8*^*-/-*^ cells (Fig 7F and 7H, compare blue and red lines). Together, these data indicate that infection with Mm induces necrosis in BMDMs, and that imatinib limits necrosis in a manner that depends upon CASP8.

### Imatinib-mediated reduction in pathology of granulomatous lesions is abrogated in mice lacking CASP8

**Fig 8:**
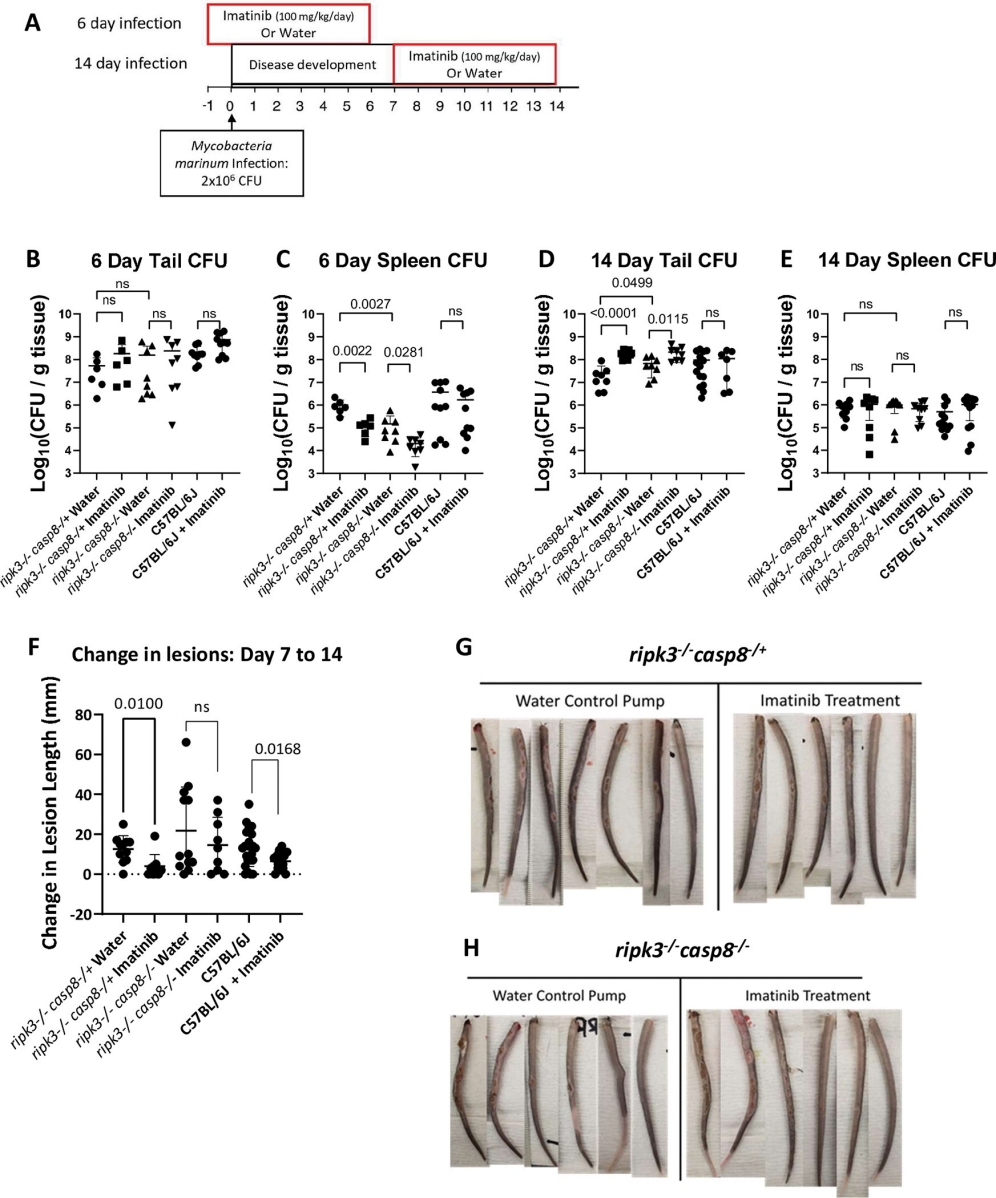
Imatinib-mediated reduction in pathology of granulomatous lesions is abrogated in mice lacking caspase 8. **A.** *ripk3*^*-/-*^ *casp8*^*-/-*^ mice or *ripk3*^*-/-*^ *casp8*^*-/+*^ mice were infected with 2x10^6^ CFU of *Mm*, for a total of 6 or 14 days. Mice were either treated with imatinib (100mg/kg/day) or water starting at day -1 or day 7 p.i. for a total of seven days, before mice were sacrificed at day 6 or 14 (n = 6–12 mice/group). **B.** Tail CFUs at the 6 day time point from the *ripk3*^*-/-*^ *casp8*^*-/+*^ and *ripk3*^*-/-*^ *casp8*^*-/-*^ treated with water or imatinib and historical controls from C57BL/6J mice infected and treated in the same manner (from Fig 1). **C.** Spleen CFUs at the six day time point from the *ripk3*^*-/-*^ *casp8*^*-/+*^ and *ripk3*^*-/-*^ *casp8*^*-/-*^ treated with water or imatinib and historical controls from C57BL/6J mice infected and treated in the same manner. **D.** Tail CFUs at the 14 day time point from the *ripk3*^*-/-*^ *casp8*^*-/+*^ and *ripk3*^*-/-*^ *casp8*^*-/-*^ treated with water or imatinib and historical controls from C57BL/6J mice infected and treated in the same manner. **E.** Spleen CFUs at the 14 day time point from the *ripk3*^*-/-*^ *casp8*^*-/+*^ and *ripk3*^*-/-*^ *casp8*^*-/-*^ with no treatment or with imatinib treatment and historical control from C57BL/6J mice infected and treated in the same manner. **F.** Lesions were measured at the beginning of treatment and at the end of treatment to determine the change in lesion size over the treatment period. Historical data from Mm-infected C57BL/6J mice treated with water or imatinib (100 mg/kg/day) was included for comparison. **G,H.** Representative images of the tail lesions from *ripk3*^*-/-*^ *casp8*^*-/+*^ **(G)** and *ripk3*^*-/-*^ *casp8*^*-/-*^ **(H)** mice. Statistical test used was two-tailed Mann-Whitney U test, with p values indicated. p values > 0.05 were considered not significant (ns). The mean +/- SD, for each group was graphed to show variance of data.

We next determined effects of imatinib on granuloma growth in *ripk3*^*-/-*^ *casp8*^*-/+*^ and *ripk3*^*-/-*^ *casp8*^*-/-*^ mice infected with Mm and exposed to imatinib or water for 7 days either starting day -1, or day 7 p.i., for a total infection period of 6 or 14 days (Fig 8A). At 6 days p.i the bacterial burden in the tails was not changed with imatinib treatment (Fig 8B), an effect evident in the C57BL/6J mice. Spleens of the knockout mice contained lower bacterial levels compared to the C57BL/6J mice, though this was not significant due to the variation evident the in the C57BL/6J animals (Fig 8C). However, the *ripk3*^*-/-*^ *casp8*^*-/-*^ developed ~ 1 log_10_ less CFU per gram of tissue than the and *ripk3*^*-/-*^ *casp8*^*-/+*^ animals. Despite this, imatinib treatment lowered spleen CFU in the of both *ripk3*^*-/-*^ *casp8*^*-/+*^ and *ripk3*^*-/-*^ *casp8*^*-/-*^ animals by ~1 log_10_ (Fig 8C). At 14 days p.i. the bacterial burden in the tails of both *ripk3*^*-/-*^ *casp8*^*-/+*^ and the *ripk3*^*-/-*^ *casp8*^*-/-*^ mice was ~7 Log_10_ CFU per gram of tissue, and imatinib treatment resulted in a 1 log_10_ (Fig 8D) increase in both genotypes. Notably, bacteria load in spleen was similar between C57BL/6J, *ripk3*^*-/-*^ *casp8*^*-/+*^, and the *ripk3*^*-/-*^ *casp8*^*-/-*^ mice, and imatinib did not cause a detectable change (Fig 8E). These data suggest that imatinib may regulate bacterial burden in manner that depends on RIPK3, reducing burden in spleen early during infection but increasing it in the tail later in infection.

At 6 days p.i. no lesions presented on any of the knockout mice, unlike the C57BL/6J mice where ~40% of untreated mice developed lesions by this time (Fig 1I). Lesion growth in water-treated *ripk3*^*-/-*^ *casp8*^*-/+*^ and *ripk3*^*-/-*^ *casp8*^*-/-*^ mice was not significantly different from that seen in C57BL/6J mice over the 7 to 14 day time period (Fig 8F). Lesions in *ripk3*^*-/-*^ *casp8*^*-/+*^ mice treated with water grew an average of 13.5mm from day 7 to 14 p.i, whereas lesions from mice treated with imatinib grew on average by 4mm over the same period (Fig 8F and 8G). Imatinib treatment reduced lesion growth by a similar amount in both the C57BL/6J and *ripk3*^*-/-*^ *casp8*^*-/+*^ animals (Fig 8F and 8G). In the *ripk3*^*-/-*^ *casp8*^*-/+*^ mice, the reduction in lesion size was evident despite an increase in CFUs in the tail the 14 day timepoint. Lesions in infected *ripk3*^*-/-*^ *casp8*^*-/-*^ mice grew by 23mm on average from day 7 to 14, with half the animals developing severe and extensive lesions measuring >30mm. Treatment of the *ripk3*^*-/-*^ *casp8*^*-/-*^ mice with imatinib did not significantly reduce lesion growth (Fig 8F and 8H). These data indicate that deletion of RIPK3 does not preclude imatinib from limiting the pathology associated with granulomatous lesions; moreover, the capacity of imatinib to limit pathology of granulomatous lesions resulting from Mm infection was abrogated in mice lacking CASP8.

Levels of TNF, IL12P70, IL10, MIP1α, Il2, IL4, IL6, GM-CSF, IL1β, and IFNγ were measured at the 6 day (S4A–S4J Fig) and 14 day (S4K–S4T Fig) timepoints in *ripk3*^*-/-*^ *casp8*^*-/+*^ or *ripk3*^*-/-*^ *casp8*^*-/-*^ animals. At 6 days p.i. imatinib did not significantly alter levels of cytokine or chemokines produced in either genotype, however, levels of IL2 (S4E Fig), GM-CSF (S4H Fig), and IFNγ (S4J Fig) were significantly lower in the *ripk3*^*-/-*^ *casp8*^*-/-*^ animals compared to the *ripk3*^*-/-*^ *casp8*^*-/+*^ animals. At 14 days p.i., no significant changes in cytokine or chemokine levels were evident between genotypes (S4K–S4T Fig). However, in the *ripk3*^*-/-*^ *casp8*^*-/-*^ mice levels of MIP1α (S4N Fig) and IL4 (S4P Fig) were increased with imatinib, and in the *ripk3*^*-/-*^ *casp8*^*-/+*^ mice, imatinib reduced levels of IL1β (S4S Fig). These data are in concordance with cytokine data from C57BL/6J mice (Fig 5), and suggest that the reduction in pathology with imatinib is not driven by changes in cytokines or chemokines.

## Discussion

### Imatinib augments anti-mycobacterial immune responses

Imatinib both induces myelopoiesis and promotes phagolysosome fusion [46,48]. Data presented here indicate that many untreated mice develop transcriptional responses associated with immune activation after three weeks of infection that resemble those induced in infected animals after one week with imatinib (Fig 3). Moreover, imatinib-induced transcriptional responses at three weeks appear more uniform owing to an increased proportion of mice displaying similar responses at that time point. In short, the response to infection with imatinib is more rapid and more uniform, but not substantially different from that developing at three weeks without drug. One possible explanation for the more rapid response to infection with imatinib is an increased capacity for phagolysosomal fusion in infected macrophages [46,47], which may preclude the capacity of mycobacteria to limit macrophage activation and antigen presentation, an idea suggested recently by Ernst and colleagues [67]. Additionally, increased numbers of innate immune cells resulting from enhanced myelopoiesis with imatinib could facilitate a more robust innate response [48].

A prominent GO term describing the effects of imatinib at early and late timepoints related to immune cell activation, which includes cytokine and chemokine responses. Such responses have been implicated in Mtb and Mm infections [11,56,68–70]. For example, mice deficient in TNF exhibit disorganized granulomas [29,30], however this effect on granuloma structure has been attributed to the inability of the host to control the infection resulting in necrotic death of macrophages [71,72]. Accordingly, patients administered drugs that neutralize or inhibit TNF are at increased risk of reactivating TB [31–33]. Sustained increases in TNF are associated with chronic inflammation and tissue damage in inflammatory bowel disease and rheumatoid arthritis [59], and with ROS-mediated tissue damage in Mm infections in zebrafish [73,74]. However, other cytokines, including IL12 and interferon-γ (INF-γ), also contribute to development and organization of granulomas, and in the activation of innate immune cells that kill Mtb [34,57,69,70,75].

While cytokines signaling is upregulated by imatinib and correlated with macrophage activation (Figs 5 and 6), no particular cytokine or group of cytokines correlates with imatinib effects on granulomas. Thus, some cytokines associated with granulomas, including TNF and IL12, are induced by imatinib in C57BL/6J animals, but not in *ripk3*^*-/-*^ *casp8*^*-/+*^ animals, where imatinib-mediated reductions in granulomas are still apparent. Moreover, other key cytokines such as IFNγ are not induced by imatinib (Fig 5). Finally, imatinib effects on granulomas appeared to be independent of bacterial load. Notably, imatinib upregulates IL10, suggesting that augmented inflammatory cytokine signaling may be restricted by factors that regulate or dampen inflammation. We cannot rule out the possibility that reduced overall inflammation evident in granulomas with imatinib is associated with upregulation of such regulatory cytokines, a possibility we are currently testing. Thus, although imatinib appeared to activate both inflammatory and regulatory cytokines implicated in mycobacterial infections, these effects did not appear to be correlated with the development, growth, or maintenance of granulomas.

### Imatinib limits granuloma formation and growth

Granulomas have long been considered a beneficial response against mycobacterial infection as they limit bacterial dissemination. However, during Mtb infection, these structures have also been shown to promote chronic infection by limiting access of immune cells to bacteria [76,77]. Granulomas also limit access of antibiotics to the bacteria, resulting in suboptimal concentrations in the caseum, where most bacteria reside [39,40]. Bacterial persistence can also induce chronic inflammation, which can scar and damage lung tissue. Moreover, attempts to heal damaged tissue results in deposition of collagen, which causes fibrosis and restricts pulmonary elasticity [78].

We have shown previously, using a low Mm inoculum and a pretreatment protocol that imatinib reduced both lesion formation and CFUs, particularly in the spleen [46]. In this study, we sought to assess transcriptional effects correlated with lesion size, and to assess effects of imatinib on lesions that had already formed, conditions that would be more reflective of those encountered in treating human TB patients. As such, we ensured generation of lesions at high frequencies by using a higher inoculum of bacteria. We were cognizant that the higher inoculum might mitigate effects of imatinib both on initial lesion formation and on CFU, but nevertheless might maximize the signal to noise across animals *vis a vis* transcriptional changes, and therefore allow identification of mechanisms associated with pathology. Higher inoculums did indeed mask imatinib effects on CFUs, but the data also suggest that imatinib provided in a treatment context still reduces lesion size in a manner that does not depend on CFUs. Microscopic analysis indicated that imatinib reduced the amount of inflammation but not the composition as necrosis, calcification, edema, ulceration, thombi, acute inflammation, and chronic inflammation were observed in both groups (Fig 2). Taken together, these data indicate that imatinib reduces the growth of the lesions and the extent of inflammation but does not otherwise alter histological features of the lesions.

Imatinib has been shown to limit collagen deposition by inhibiting PDGFR and fibrosis in non-infectious indications [50–55]. Accordingly, reductions in collagen are suggested by the GO analysis of gene expression data from imatinib-treated mice (Figs 4 and S2). These data raise the possibility that imatinib may facilitate wound healing in a way that is less likely to induce fibrosis, which in turn may limit scaring and long-term tissue damage, a prospect we are currently testing. This finding has important implications for lung health during Mtb infection. Chronic inflammation has been associated with increased fibrosis and lung dysfunction in TB patients [38,41,42,49], which results in increased economic burden of patients who have successfully completed antibiotic therapy [44]. We postulate that imatinib may be an effective treatment for patients with active TB by reducing fibrosis and lung dysfunction. The structure of the granuloma has also been shown to limit antibiotic penetrance into the granuloma [39,40]. By limiting formation and/or promoting resolution of granulomas, imatinib may increase access of antibiotics to the bacteria and thereby shorten duration of treatment.

### Imatinib promotes cell survival and limits necrosis

Cell death is a major contributing factor to bacterial spread and tissue pathology [34,79–81]. Apoptosis has been shown to limit spread and replication of Mtb by encapsulating the bacteria in apoptotic bodies and allowing macrophages to efferocytose apoptotic bodies containing Mtb, and transfer them into the lysosome [82]. By contrast, necrosis facilitates spread of bacteria, promotes inflammation, and contributes to tissue damage by releasing ROS [34,81,83]. Ferroptosis, an iron-dependent form of necrosis, has recently been identified as a major contributor to pathology during Mtb infection [83]. Several lines of evidence indicate that altering the type of death that a cell undergoes can impact Mtb infection. For example, a mutation in the Lpr1 gene, which encodes LDL receptor, can shift macrophages from necrosis to apoptosis, thereby limiting replication and spread of the bacteria [80]. In this regard, imatinib has been shown to increase apoptotic cell death in BCR-Abl^+^ transformed cells but is generally without effect on non-transformed cells [84], an effect we observed in uninfected BMDMs (Fig 7). Tails in mice infected with Mm display reduced gross pathology and more focused areas of tissue damage with imatinib, which is consistent with reductions in overall levels of necrosis and/or increased cell survival. No quantitative changes in necrosis at a gross histological level were evident (S1 Fig), though our measurements were made on tissue sections selected because they contained granulomas. In cultured macrophages, imatinib has little effect on uninfected cells, but reduced necrosis in infected ones (Fig 7).

The impact of CASP8 *in vivo* can only be assessed in the context of a RIPK3 deficiency because knocking out CASP8 alone results in embryonic lethality [66]. Our data with the *ripk3*^*-/-*^ *casp8*^*-/-*^ mice suggest a possible role for CASP8 in imatinib effects on granulomas (Figs 7 and 8), possibly by regulating necrosis, or cell survival, or both. In this regard, CASP8 promotes extrinsic apoptosis in response to signaling via death receptors such as TNF receptor 1 and CD95 [59,85], though no evidence of CASP8-mediated apoptosis was evident in our analysis (Fig 7A). However, CASP8 also promotes cell survival by complexing with c-FLIP, which inhibits apoptosis, and precludes activation of RIPK3, thereby preventing necroptosis [61,63]. Finally, CASP8 regulates transcriptional responses that dampen inflammation [86]. Any of these activities could contribute to imatinib’s effect on reducing granuloma growth or limiting inflammation *in vivo*. Further characterization of cellular mechanisms associated with enhanced survival and/or reduced necrosis with imatinib and CASP8 are being pursued to elucidate how the drug facilitates resolution of granulomas. Notably, both the *ripk3*^*-/-*^ *casp8*^*-/-*^ mice and the control *ripk3*^*-/-*^ *casp8*^*-/*+^ mice are deficient in necroptosis. Despite this, differences in pathology are only evident when comparing imatinib effects in the *ripk3*^*-/-*^ *casp8*^*-/*+^ mice; thus, pathological differences with imatinib appear to be independent of RIPK3, and necroptosis does not appear to play a role. A more complete characterization of effects of imatinib on other forms of necrosis is in progress.

In mice infected with Mtb, it has been suggested that RIPK3 facilitates the spread of bacteria [87] by mediating necrotic death, and mice lacking RIPK3 developed lower bacterial CFUs in their lungs. Interestingly, CFUs in the tails of *ripk3*^*-/-*^ *casp8*^*-/*+^ or *ripk3*^*-/-*^ *casp8*^*-/-*^ animals increased with imatinib at 14 days p.i.. Whereas this increase was significant with imatinib treatment in each mouse genotype, it did not reach significance when compared to CFUs in C57BL/6J animals. However, despite this increase in tail CFUs, no differences in spleen CFUs were apparent at this timepoint (Fig 8). Moreover, at 6 days p.i. in the *ripk3*^*-/-*^ *casp8*^*-/*+^ or *ripk3*^*-/-*^ *casp8*^*-/-*^ animals, imatinib reduced CFUs in the spleens of both RIPK3 genotypes by ~1 order of magnitude, despite a lack of effect in the tails (Fig 8). These data raise the possibility that RIPK3 and/or necroptosis may mediate imatinib effects on either bacterial dissemination, or bacterial killing, or both.

### Imatinib as an HDT

Recent efforts by us and others [88] have raised the possibility that HDTs may be effective against Mtb as well as Mm. Because HDTs do not directly select against the bacteria, they are less likely to engender resistance compared to antibiotics. Some HDTs interfere with the capacity of Mtb to subvert host systems, whereas others regulate immune responses, so as to induce novel responses or target deficiencies in individuals with active disease [89]. Drug discovery efforts have traditionally focused on drugs targeting specific processes with minimal off-target effects, though recent efforts, for example with COVID-19, have targeted hyperinflammatory pathways as a means to limit tissue damage [89]. Such approaches can be complex to implement, requiring proper timing and dosing to mitigate tissue damage while still permitting requisite inflammatory responses that contain the infection. Our studies suggest that imatinib likewise affects multiple aspects of the host immune response to mycobacteria, but it does so in ways that do not fundamentally alter the response, but rather tunes it to favor the host by augmenting the rate at which it develops.

Imatinib remains a promising candidate HDT to treat mycobacterial infections such as TB and NTM by not only activating innate immune responses but now also reducing pathology. Imatinib is currently being tested in the IMPACT-TB clinical trial to determine effective dosing in healthy humans as measured by the capacity of human blood to eliminate mycobacteria. Data presented here shows how an HDT can limit granuloma formation. Such an effect has important implications for therapeutic strategies to control TB as the granuloma has been shown to limit antibiotic and immune cell access to the bacteria, leading to bacterial persistence and contributing to lasting tissue damage and scaring.

## Materials and methods

### Ethics statement

All procedures performed on mice were approved by the Institutional Animal Care and Use Committee (IACUC) of Emory University (Protocol PROTO202000158). Mice were bred and maintained by Emory University Division of Animal Resources in accordance with the NIH Guide for the Care and Use of Laboratory Animals. All animal infections were conducted under animal biosafety level (ABSL) 2.

### Mice

8–12-week-old C57BL/6J mice were purchased from The Jackson Laboratory. *ripk3*^*-/-*^ *casp8*^*-/*-^, or *ripk3*^*-/-*^ *casp8*^*-/*+^ were bred at Emory University by crossing *ripk3*^*-/-*^ *casp8*^*-/+*^ females to *ripk3*^*-/-*^ *casp8*^*-/*-^ males [66]. PCR genotyping of *casp8*^−/−^ and *casp8*^−/+^ mice was performed with primers 5′-TTGAGAACAAGACCTGGGGACTG and 5′-GGATGTCCAGGAAAAGATTTGTGTC. PCR amplification allele produces a 750-bp band (*casp8*^+^), or a 200-bp band (*casp8*^−^).

### Imatinib administration

Imatinib mesylate salt was dissolved in water and loaded into Alzet pumps (Braintree Scientific, 1007D; Cupertino, CA) capable of dispensing a continuous flow of drug at 100mg/kg/day. Pumps were inserted subcutaneously into anesthetized 8-12-week old mice. Alzet pumps were inserted 24 h prior to infection or 7–21 days post infection depending on experiment time course.

### Bacterial strains

*M*. *marinum (*Mm*)* strain 1218R (ATCC 927) was grown in Middlebrook 7H9 broth (7H9) (BBL Microbiology Systems, Cockeysville, MD) supplemented with ADC (Difco Laboratories, Detroit, MI,) and 0.05% Tween 80 (Sigma-Aldrich, St. Louis, MO). For CFU assays, 7H10 agar supplemented with 10% oleic acid-albumin-dextrose-catalase (OADC) was used (Difco Laboratories, Sparks, MD). For Mm infection in mice and infections of BMDMs, bacterial stocks were grown at 30°C for 2 days to an OD_600_ of 0.4 approximately 6.3x10^6^CFU/mL (Eppendorf, BioPhotometer; Hamburg, Germany). Bacteria were washed with sterile PBS and aspirated through a 27G needle against the side of the tube multiple times to break up bacterial clumps. Bacteria was then diluted in sterile phosphate buffered saline [PBS] for mouse infection or complete cell culture media for the BMDM infections.

### Mouse Mm infection

Mice were infected with actively growing Mm (ATCC 927) via the tail vein to induce a systemic infection that results in the development of mycobacterial granulomas on the tail of the mouse. The inoculum, determined by retrospective plating, was ~2x10^6^CFU in each experiment. In C57BL/6J mice tail lesions appear on the mouse tail about 1 week after infection and can be monitored over time by measuring the length of each lesion from top to bottom using a ruler, to get a measure of the total lesion lengths per mouse tail. After 7 days of treatment, tail, spleen, and lung were harvested. For CFU, spleens, and lung, were weigh and homogenized in the Bullet Blender Tissue homogenizer (Next Advance, Troy, NY) in 1 ml PBS. ~3–5 mm of tail was cut into small pieces using sterile scissors before being homogenized in the same way as lung and spleen. Each homogenate was diluted and spread on 7H10 agar plates. Colonies were scored after 7 days at 30°C. Colonies per ml were normalized to the initial weight of the tissue, to determine CFU/g tissue.

### Tail protein isolation for ELISA and Western analysis

For soluble protein isolation for ELISA, 3-5mm sections of tail were cut fresh from the tail and immediately placed in liquid nitrogen. Samples were stored at -80°C until processed. Samples were weighted and put in PBS, scissors were used to break apart the tissue before the tissue was homogenized in the Bullet Blender Tissue homogenizer (Next Advance, Troy, NY). IL12P70 (Invitrogen), TNFα (Invitrogen), and IL10 (Invitrogen) ELISAs were run according to manufacturer’s directions. For western analysis of tail protein content, 3–5 mm of flash frozen tail samples were weighted and put in ice cold RIPA buffer supplemented with a protease inhibitor cocktail (Roche). Scissors were used to break apart the tissue before the tissue was homogenized in the Bullet Blender Tissue homogenizer (Next Advance, Troy, NY). Protein concentration was determined using BCA assay kit (ThermoFisher), before the samples were run on 15% SDS-PAGE gel and blotted onto a PVDF membrane. Cleaved Caspase 3 (ASP175) antibody (1:1000, Cell Signaling) and GAPDH (D16H11) antibody (1:1000, Cell Signaling) were used sequentially on the same membrane to quantify and normalize protein content.

### Multiplexed cytokine quantification

Ten immune mediators, including nine cytokines (TNF, IL12P70, IL10, IL2, IL4, IL6, GM-CSF, IL1β, and IFNγ) and one chemoattractant (MIP1α), were measured in soluble tail protein samples using a multiplexed chemiluminescent assay (U-PLEX Custom Biomarker Group; Meso Scale Diagnostics) in accordance with manufacturer’s protocols. A QuickPlex SQ 120 was used to acquire data and Discovery Workbench V4 (Meso Scale Diagnostics) was used for analysis. If a value was below the lower limit of detection, a value of half the lower limit of detection for that specific analyte in that assay was imputed.

### Mouse tail RNA isolation

3-5mm sections of tail were cut fresh from the tail from areas containing granulomatous lesions or near the injection site for uninfected mice or mice with no visible lesions and immediately placed in Trizol (Invitrogen). Tissue was cut with sterile scissors and then homogenized in the Bullet Blender Tissue homogenizer (Next Advance, Troy, NY). RNA extraction was performed in accordance with Invitrogen protocols.

### Histology

Tails were removed from the mouse and a clean razor used to slice tails into 3–5 mm lengths that were placed in a cassette and submerged in 10% neutral buffered formalin for 24–48 hours. Tail sections were washed and decalcified in Immunocal decalcifier (StatLab, McKinney, Tx) for 48 hrs before being moved to 70% ethanol for storage until the sections could be embedded in paraffin, and sectioned. Sections were stained by H&E (Abcam) and acid-fast bacillus [AFB] stain (Abcam).

### Histology scoring

Histology was obtained from the tails of mice infected for 14 days and treated with control or 100 mg/kg/day imatinib for the last 7 days of infection. Slides of H&E-stained tail sections containing 2–9 individual tail tissue cross sections/mouse were deidentified and randomized before they were submitted to a pathologist for scoring. One of us (Guarner) examined slides first to create a rubric for characteristics, then blindly scored all the sections based on the noted characteristics. The percent of inflammation was determined in the section with highest amount of inflammation.

### BMDM generation

L929 cells were maintained in DMEM (Corning) with 10% fetal bovine serum (FBS, Gibco), 100U/ml penicillin and 100 U/ml streptomycin (Invitrogen). At confluency, fresh media was added to the L929 cells and collected and sterile filtered after 3 days. L929 conditioned media was stored at -20°C until use. For BMDM culture, tibias and femurs were collected from C57BL/6J, *ripk3*^*-/-*^ *casp8*^*-/*-^, or *ripk3*^*-/-*^ *casp8*^*-/*+^ mice. The ends of the bones were removed with a clean razor before the bones were placed in a sterile 0.7 ml Eppendorf tube with the bottom tip cut off inside a 1.5 mL sterile Eppendorf tube. Tubes were centrifuged at 13,000 RPM for 3 minutes to extrude the bone marrow cells. Cells were washed and filtered through a 70μM mesh filter with sterile PBS, before being plated in 10 cm petri dishes with 20% L929 conditioned media in fresh DMEM + 10% FBS + 100U/ml penicillin and 100 U/ml streptomycin. Cells were differentiated for 7–9 days before collection in ice cold PBS containing 0.5M EDTA.

### BMDM RNA and protein collection

For RNA and protein collection, BMDMs were plated at 1x10^6^ cells/ well in a 6 well plate. Cells were allowed to adhere overnight before being infected with Mm at an MOI of 10 and treated with 1μM imatinib. Infection commenced for 24 hrs, at which time media was removed and stored at -80°C for cytokine quantification. TNFα (Invitrogen), and IL10 (Invitrogen) ELISAs were performed according to manufacturer’s protocols on undiluted media. Cells were lysed and RNA was collected using the RNeasy Mini Kit (Qiagen), according to manufacturer’s protocols.

### BMDM cell viability assay

For cell viability assays, 10,000 cells/well were plated into a 96-well plate and allowed to adhere overnight. Cells were infected with actively growing Mm at an MOI of 5 and treated with 1 μM imatinib in DMEM + 10% FBS and placed in an incubator at 37°C with 5% CO2. After 20 hours of infection, Cell Titer-Blue (Promega, Madison, WI) was added to the media and cells were incubated for 4 hrs at 37°C with 5% CO2 before fluorescence was measured using a plate reader.

### BMDM Time course experiments

For time course experiments, 1-3x10^5^ cells/well were plated into a 24 well plate and allowed to adhere overnight. BMDMs from C57BL/6J mice were infected with actively growing Mm at an MOI of 1 of while BMDMs from *ripk3*^*-/-*^ *casp8*^*-/*-^ and *ripk3*^*-/-*^ *casp8*^*-/*+^ mice were infected with actively growing Mm at an MOI of 10 for 2 hrs in DMEM + 10% FBS. Wells were washed with PBS and 200 μg/ml amikacin in DMEM + 10% FBS was added for 2 hrs. Cells were washed again with PBS before DMEM + 10% FBS was added with 250nM Incucyte Cytotox Green (Sartorius) +/- 1 μM imatinib. Trays were then placed in the IncuCyte ZOOM (Saritorius Essen) at 37°C with 5% CO_2_. Phase images and Green Fluorescence Images were taken at 20x every 2 hrs. The area of phase objects and the area of green objects (necrotic cells) were quantified to determine the green area per phase area for each image, a number indicating the amount of necrosis.

### RNA-seq analysis

RNA was isolated from mouse tails from uninfected mice +/- 100mg/kg/day imatinib, infected mice at 6 days post infection +/- 100mg/kg/day imatinib, or infected mice 21 days post infection +/- 100mg/kg/day imatinib. RNA from the tails of 5 individual mice were used per group for a total of 30 samples. RNA libraries for RNA-seq were prepared using Clontech SmartER Stranded Total RNA-seq Kit- Pico Input Mammalian + rRNA depletion following manufacturer’s protocols. Sequencing was performed at Yerkes Nonhuman Primates Genomics Core, Emory University, using Illumina NovaSeq 6000. Quality control was performed on the raw reads using FastQC and the remaining analysis was done using R. Adaptors were trimmed from the ends of reads using QuasR. Hisat2 was used for alignment of the reads to the GRCm38.p6 mouse genome with 66–80% of reads mapped to a single gene. 6–25% of the mapped reads were successfully assigned to gene alignments resulting in 5.5 million-14million assigned alignments per sample. Genes with low expression levels (>20 copies per million) in at least 5 samples filtered out. Data was normalized using the Trimmed Mean of M-values [TMM] method. Differential expression analysis was performed using edgeR, genes were determined by comparison of each group to the uninfected mice not treated with imatinib. Raw reads and processed gene counts in this paper have been deposited in the Gene Expression Omnibus (GEO) database, https://www.ncbi.nlm.nih.gov/geo/(accession no. **GSE215176**).

### QPCR

RNA was isolated from mouse tail or BMDMs as previously described. Reverse transcription was performed using the RevertAid First-Strand cDNA Synthesis Kit (Thermo Fisher Scientific) with the oligo(dT)18 primer. iQ SYBR Green Supermix (Bio-Rad) according to manufacturer instructions using a MyiQ real-time PCR system (Bio-Rad). The ΔΔCT method was used to determine relative gene expression using *Gapdh* as internal controls. Primers used were: *Gapdh-*forward 5’ AGGTCGGTGTGAACGGATTTG3’, *Gapdh-*reverse 5’TGTAGACCATGTAGTTGAGGTCA3’, *il12b*-forward 5’TGGTTTGCCATCGTTTTGCTG3’, *il12b*-reverse 5’ACAGGTGAGGTTCACTGTTTCT3’, *tnf-*forward 5’CCCTCACACTCAGATCATCTTCT3’, *tnf-*reverse 5’ GCTACGACGTGGGCTACAG3’, *il10-* forward 5’GCTCTTACTGACTGGCATGAG3’, *il10-* reverse 5’CGCAGCTCTAGGAGCATGTG3’, *nos2-*forward 5’ GTTCTCAGCCCAACAATACAAGA3’, *nos2-*reverse 5’ GTGGACGGGTCGATGTCAC3’, *chil3l3-*forward 5’CAGGTCTGGCAATTCTTCTGAA3’, *chil3l3-*reverse 5’GTCTTGCTCATGTGTGTAAGTGA3’, *arg1*-forward 5’CTCCAAGCCAAAGTCCTTAGAG3’, *arg1*-reverse 5’AGGAGCTGTCATTAGGGACATC3’. The date generated was normalized to the lowest value.

### Statistical analysis

Statistical analysis was done using either Mann-Whitney U test to compare two groups or a one-way Kruskal-Wallis or ANOVA to compare multiple groups. Values less than or equal to 0.05 were considered statistically significant. For RNA-seq analysis the false discovery rate (FDR) of less than 0.05 was used to determine differentially expressed genes and GO processes.

## Supporting information

S1 Fig Histology measurements of mouse tails. C57BL/6J mice were infected with 2x10^6^ CFU of *Mm*. Beginning at day 7 p.i., mice were treated with imatinib at 100mg/kg/day or water for 7 days. Cross sections of the tail were cut and stained with H&E (A-N) or acid-fast bacillus (AFB) stain. **A.** Representative H&E images showing bone marrow necrosis and inflammation, and muscle thrombosis and necrosis at a magnification of 200x. **B-G.** Pathology scores for the epidermis and dermis for ulceration **(B)**, necrosis **(C)**, calcification **(D)**, edema **(E)**, acute inflammation **(F)**, and chronic inflammation **(G)**. **H-L.** Pathology scores for the muscle for necrosis **(H)**, edema **(I)**, thrombi **(J)**, acute inflammation **(K)**, and chronic inflammation **(L)**. **M,N.** Pathology scores for the bone for necrosis **(M)**, and inflammation **(N)**. **O.** Representative images of AFB stain in the mouse tail lesions with micro-organisms identified in circles at a magnification of 1000x. Each data point represents scoring from one individual mouse in 4 separate experiments (n = 21/group). Statistical test used was two-tailed Mann-Whitney U test, with p values indicated. p values >0.05 were considered not significant (ns). The mean +/- SD, for each group was graphed to show variance of data.
(TIF)

S2 Fig GO analysis of imatinib induced and infection related genes. **A,B.** Genes differentially expressed with imatinib treatment in the absence of infection were identified by comparing differentially expressed genes from the uninfected water group, to imatinib-treated mice (514 genes, FDR < 0.05). Selection of GO terms identified by GO analysis of 373 genes upregulated with imatinib treatment (**A).** Selection of GO terms identified by GO analysis of 141 genes downregulated with imatinib treatment (**B). C,D.** Selection of GO terms identified by GO analysis of the 903 genes upregulated at the 1 week infection timepoint **(C)** and the 676 genes upregulated at the 3 week infection timepoint **(D)** identified in Fig 3B. **E.** Comparison of **“**Infection Genes” (green circle; genes identified in Fig 3B), imatinib genes at 1 week infection (red circle; genes identified in Fig 3B), and imatinib genes at 3 weeks of infection (yellow circle; genes identified in Fig 3C).
(TIF)

S3 Fig Imatinib effects on BMDM during Mm infection. **A.** RNA was collected from BMDMs derived from C57BL/6J mice infected with Mm at an MOI of 10 and left untreated or treated with 1μM imatinib for 8 hrs, 24 hrs, or 44 hrs. qPCR was used to measure levels of *nos2* in the cells at each time point (n = 2 wells/ group; data are representative of 3 separate experiments). **B-D.** BMDMs derived from C57BL/6J mice infected with Mm at an MOI of 10 and left untreated or treated with 1μM imatinib for 24 hrs. RNA was isolated from the cells and qPCR was used to measure levels of *nos2* **(B)**, *chil3l3* **(C)**, and *arg1* **(D;** n = 6 wells/ group). Statistical test used was one-way ANOVA, with p values indicated. p values >0.05 were considered not significant (ns). The mean +/- SD, for each group was graphed to show variance of data.
(TIF)

S4 Fig Cytokine changes with imatinib at 6 and 14 days post infection in *ripk3^-/-^ casp8^-/+^* and *ripk3^-/-^ casp8^-/-^* mice. *ripk3*^*-/-*^ *casp8*^*-/-*^ mice or *ripk3*^*-/-*^ *casp8*^*-/+*^ mice were infected with 2x10^6^ CFU of *Mm*, for a total of 6 or 14 days. Mice were either treated with imatinib (100mg/kg/day) or water starting day -1 or day 7 relative to the start of infection, for a total of 7 days before mice were sacrificed at day 6 or 14 (n = 6–12 mice/group). Protein was isolated from tail sections with visible lesions or near the injection site to measure cytokine and chemokine content. **A-J**. At 6 days p.i., TNF **(A)**, Il12p70 **(B)**, Il10 **(C),** MIP1α **(D)**, IL2 **(E)**, IL4 **(F)**, IL6 **(G)**, GM-CSF **(H)**, IL1β **(I)**, and IFNγ **(J)** were measured via a multiplexed chemiluminescent assay. **K-T**. At 14 days p.i., TNF **(K)**, Il12p70 **(L)**, Il10 **(M),** MIP1α **(N)**, IL2 **(O)**, IL4 **(P)**, IL6 **(Q)**, GM-CSF **(R)**, IL1β **(S)**, and IFNγ **(T)** were measured via a multiplexed chemiluminescent assay. Statistical test used was Kruskal-Wallis, with p values indicated. p values > 0.05 were considered not significant (ns). The mean +/- SD, for each group was graphed to show variance of data.
(TIF)
