# Supplementary figures and images for: The host-directed therapeutic imatinib mesylate accelerates immune responses to Mycobacterium marinum infection and limits pathology associated with granulomas
Source: PLoS Pathog. 2023 May 18;19(5):e1011387. doi: 10.1371/journal.ppat.1011387 (PMC10231790; doi:10.1371/journal.ppat.1011387)

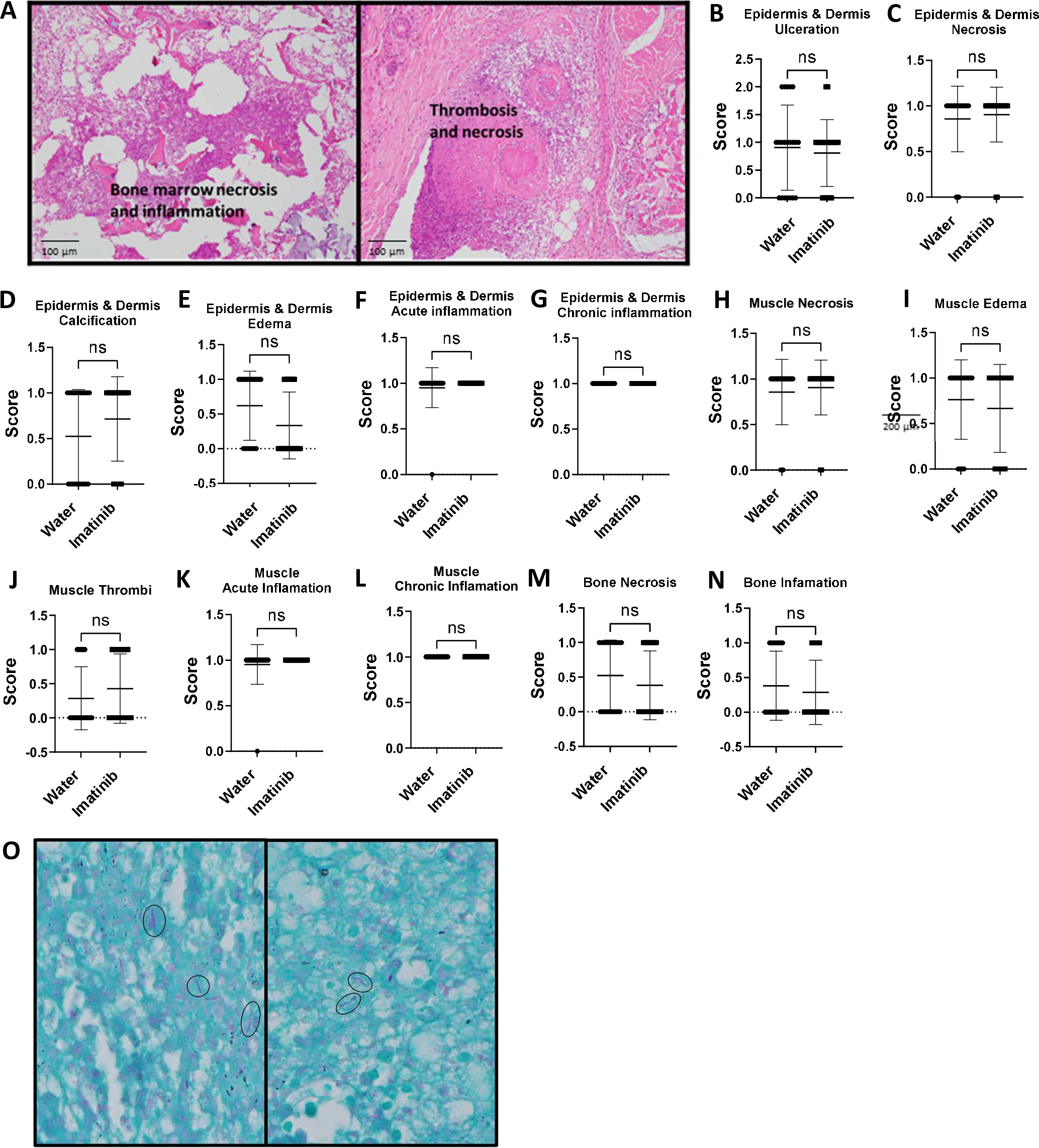

Supplement: S1 Fig — C57BL/6J mice were infected with 2x10^6^ CFU of Mm. Beginning at day 7 p.i., mice were treated with imatinib at 100mg/kg/day or water for 7 days. Cross sections of the tail were cut and stained with H&E (A-N) or acid-fast bacillus (AFB) stain. A. Representative H&E images showing bone marrow necrosis and inflammation, and muscle thrombosis and necrosis at a magnification of 200x. B-G. Pathology scores for the epidermis and dermis for ulceration (B), necrosis (C), calcification (D), edema (E), acute inflammation (F), and chronic inflammation (G). H-L. Pathology scores for the muscle for necrosis (H), edema (I), thrombi (J), acute inflammation (K), and chronic inflammation (L). M,N. Pathology scores for the bone for necrosis (M), and inflammation (N). O. Representative images of AFB stain in the mouse tail lesions with micro-organisms identified in circles at a magnification of 1000x. Each data point represents scoring from one individual mouse in 4 separate experiments (n = 21/group). Statistical test used was two-tailed Mann-Whitney U test, with p values indicated. p values >0.05 were considered not significant (ns). The mean +/- SD, for each group was graphed to show variance of data. (TIF) [file ppat.1011387.s001.tif]

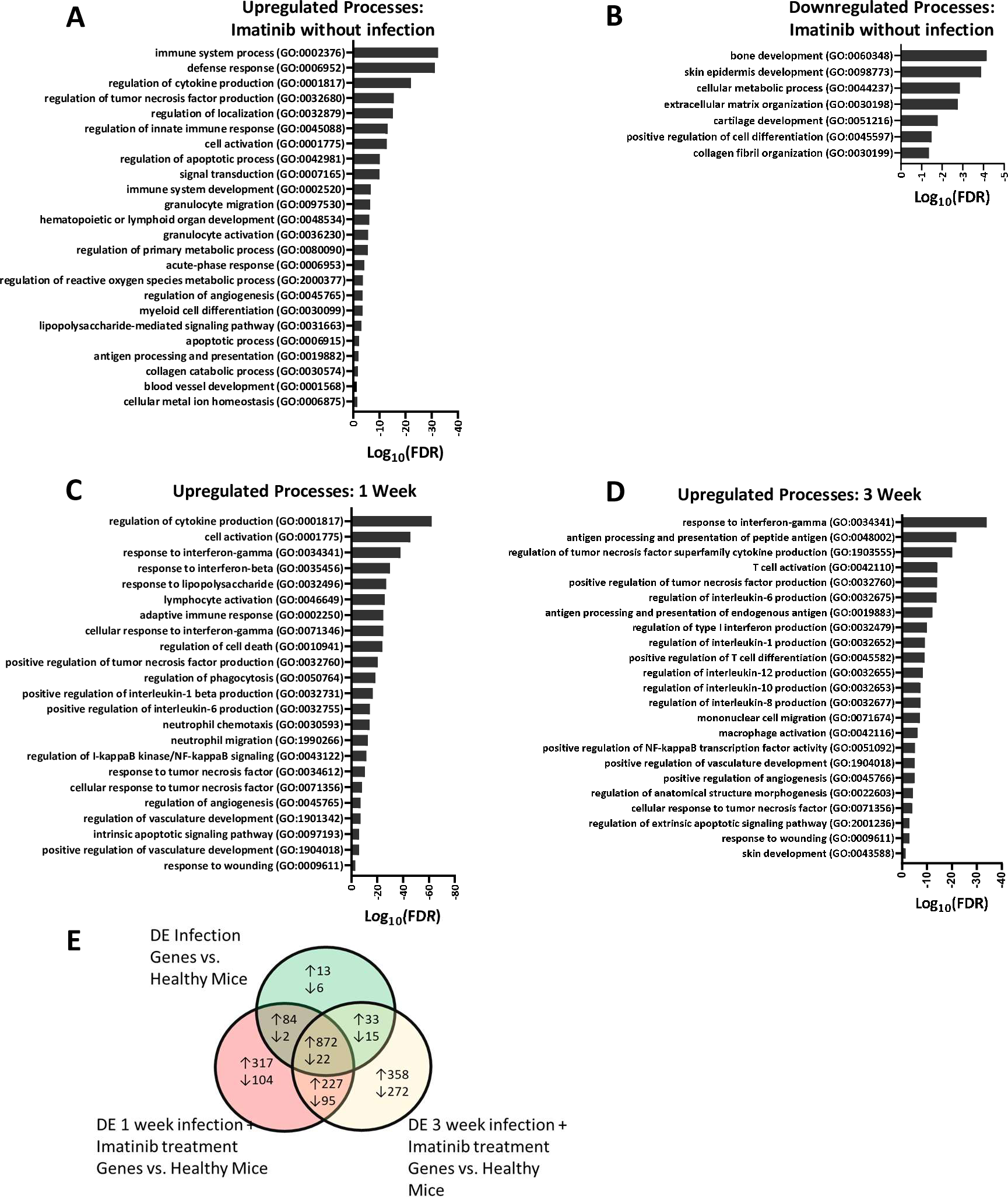

Supplement: S2 Fig — A,B. Genes differentially expressed with imatinib treatment in the absence of infection were identified by comparing differentially expressed genes from the uninfected water group, to imatinib-treated mice (514 genes, FDR < 0.05). Selection of GO terms identified by GO analysis of 373 genes upregulated with imatinib treatment (A). Selection of GO terms identified by GO analysis of 141 genes downregulated with imatinib treatment (B). C,D. Selection of GO terms identified by GO analysis of the 903 genes upregulated at the 1 week infection timepoint (C) and the 676 genes upregulated at the 3 week infection timepoint (D) identified in Fig 3B. E. Comparison of “Infection Genes” (green circle; genes identified in Fig 3B), imatinib genes at 1 week infection (red circle; genes identified in Fig 3B), and imatinib genes at 3 weeks of infection (yellow circle; genes identified in Fig 3C). (TIF) [file ppat.1011387.s002.tif]

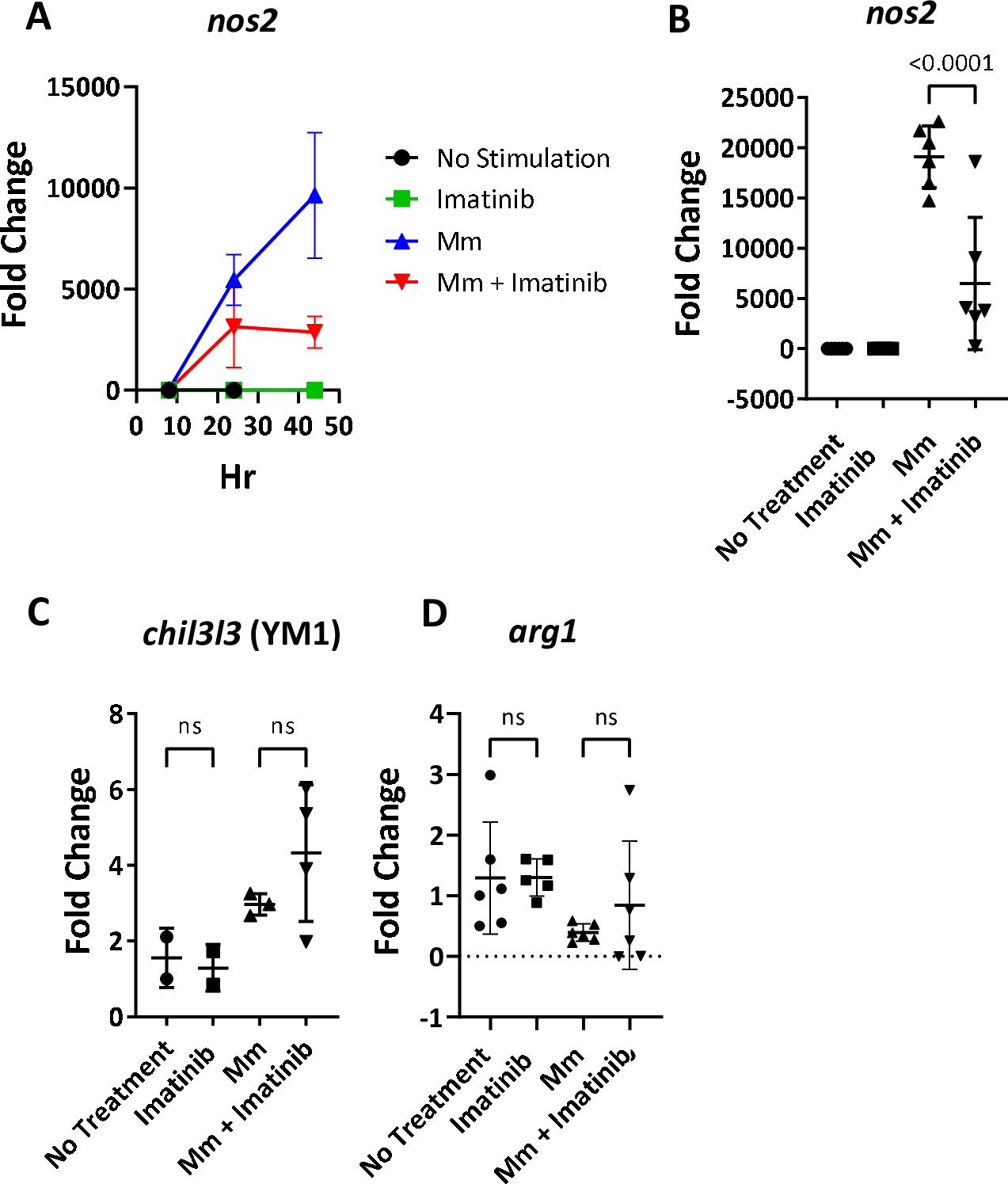

Supplement: S3 Fig — A. RNA was collected from BMDMs derived from C57BL/6J mice infected with Mm at an MOI of 10 and left untreated or treated with 1μM imatinib for 8 hrs, 24 hrs, or 44 hrs. qPCR was used to measure levels of nos2 in the cells at each time point (n = 2 wells/ group; data are representative of 3 separate experiments). B-D. BMDMs derived from C57BL/6J mice infected with Mm at an MOI of 10 and left untreated or treated with 1μM imatinib for 24 hrs. RNA was isolated from the cells and qPCR was used to measure levels of nos2 (B), chil3l3 (C), and arg1 (D; n = 6 wells/ group). Statistical test used was one-way ANOVA, with p values indicated. p values >0.05 were considered not significant (ns). The mean +/- SD, for each group was graphed to show variance of data. (TIF) [file ppat.1011387.s003.tif]

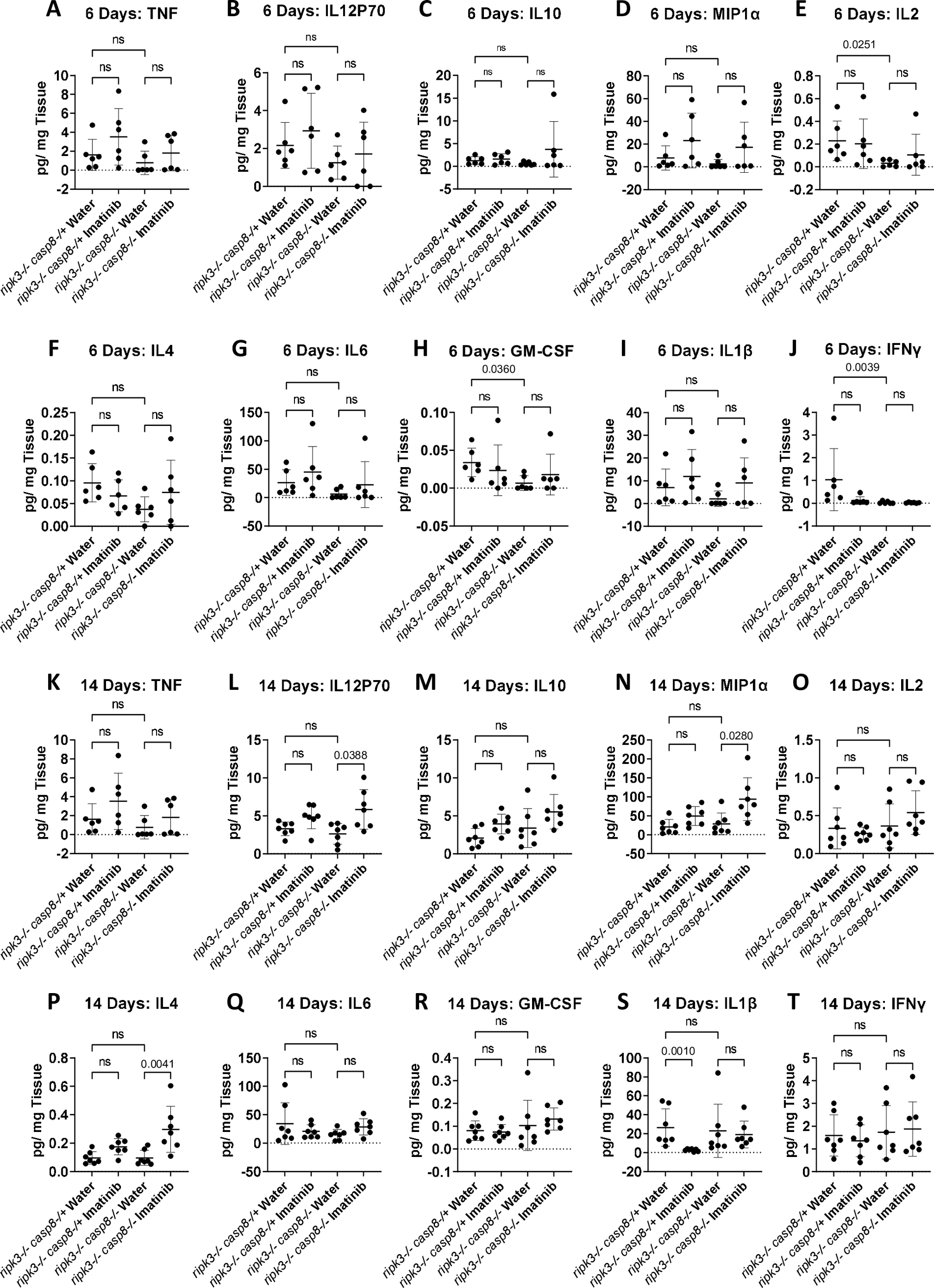

Supplement: S4 Fig — ripk3-/- casp8-/- mice or ripk3-/- casp8-/+ mice were infected with 2x10^6^ CFU of Mm, for a total of 6 or 14 days. Mice were either treated with imatinib (100mg/kg/day) or water starting day -1 or day 7 relative to the start of infection, for a total of 7 days before mice were sacrificed at day 6 or 14 (n = 6–12 mice/group). Protein was isolated from tail sections with visible lesions or near the injection site to measure cytokine and chemokine content. A-J. At 6 days p.i., TNF (A), Il12p70 (B), Il10 (C), MIP1α (D), IL2 (E), IL4 (F), IL6 (G), GM-CSF (H), IL1β (I), and IFNγ (J) were measured via a multiplexed chemiluminescent assay. K-T. At 14 days p.i., TNF (K), Il12p70 (L), Il10 (M), MIP1α (N), IL2 (O), IL4 (P), IL6 (Q), GM-CSF (R), IL1β (S), and IFNγ (T) were measured via a multiplexed chemiluminescent assay. Statistical test used was Kruskal-Wallis, with p values indicated. p values > 0.05 were considered not significant (ns). The mean +/- SD, for each group was graphed to show variance of data. (TIF) [file ppat.1011387.s004.tif]
